# Multiscale-Aware Graph Embedding Approach Uncovers LC-61, a Potent Anti-*Leishmania infantum* Compound

**DOI:** 10.1021/acs.jcim.5c02947

**Published:** 2026-03-17

**Authors:** Vinícius Alexandre Fiaia Costa, Alexandra Maria dos Santos Carvalho, Rafael Consolin Chelucci, Felipe da Silva Mendonça de Melo, Gustavo Santos Sandes Felizardo, Clarissa Alves Carneiro Bernardes, Holli-Joi Martin, Rodolpho de Campos Braga, Sébastien Charneau, Eugene N. Muratov, Adriano Defini Andricopulo, Izabela Marques Dourado Bastos, Bruno Junior Neves

**Affiliations:** † Laboratory of Cheminformatics, Faculty of Pharmacy, 67824 Federal University of Goiás, 74605-170 Goiânia, Brazil; ‡ Pathogen-Host Interface Laboratory, Department of Cell Biology, Institute of Biological Sciences, University of Brasilia, 70910-900 Brasilia, Brazil; § Laboratory of Medicinal and Computational Chemistry, Sao Carlos Institute of Physics, University of Sao Paulo, IFSC—USP, 13563-120 Sao Carlos, SP, Brazil; ∥ Laboratory for Molecular Modeling, UNC Eshelman School of Pharmacy, University of North Carolina at Chapel Hill, Chapel Hill, North Carolina 27599-7360, United States; ⊥ Laboratory of Protein Chemistry and Biochemistry, Department of Cell Biology, Institute of Biological Sciences, University of Brasilia, 70919-900 Brasilia, Brazil; # InsilicAll Ltda., 04571-010 São Paulo, Brazil

## Abstract

Visceral leishmaniasis caused by *Leishmania infantum* remains a lethal disease with few therapeutic options, necessitating innovative computational methods and approaches to accelerate drug discovery. Here, we present a graph neural network (GNN) framework incorporating well-established multiscale mechanisms to improve the identification of novel antileishmanial compounds. Across two classificatory antileishmanial data sets, our GNNs demonstrated significant improvements in predictive performance, with area under the receiver operating characteristic curve (AUC) increases of 2.2–29.2% on the imbalanced data set (activity cutoff: 1 μM) and 3.4–22.5% on the balanced data set (activity cutoff: 10 μM) compared to default GNNs. Subsequently, the framework was applied to screen a library of approximately 1.3 million compounds, pinpointing LC-61 as a potent antileishmanial agent with nanomolar activity against intracellular *L. infantum* (IC_50_ = 0.076 μM) and minimal cytotoxicity to macrophages (THP-1 CC_50_ = 157 μM). A comprehensive *in vitro* ADME profiling revealed that LC-61 combines high solubility at both acidic and physiological pH (>28 μg/mL), balanced lipophilicity (eLogD = 4.07), and favorable passive permeability (PAMPA = 4.86 × 10^–6^ cm/s), while exhibiting lower microsomal stability. Overall, our GNN framework effectively accelerated the discovery of LC-61, a novel and biologically validated hit suitable for hit-to-lead optimization.

## Graphical abstract

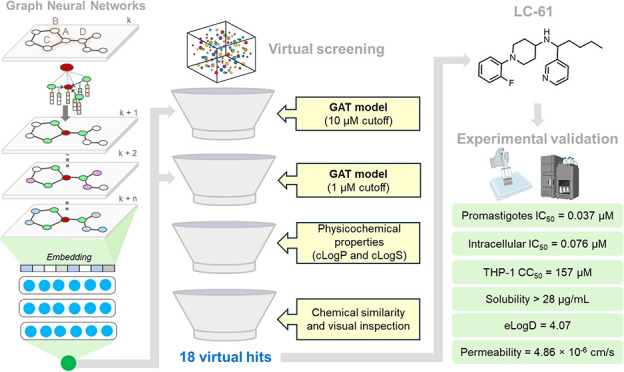

## 1 Introduction

Visceral leishmaniasis (VL) is a potentially fatal vector-borne disease transmitted by the bite of infected sandflies. It is primarily caused by two protozoan parasites of the genus *Leishmania:* *Leishmania donovani*, predominantly found in Africa and Asia, and *Leishmania infantum*, mainly present in Latin America and the Middle East.^1–3^ This disease affects vital organs such as the liver, spleen, and bone marrow, manifesting clinically as weight loss, fatigue, splenomegaly, hepatomegaly, anemia, and irregular fever.^4^ According to the World Health Organization, most cases of VL are concentrated in Brazil, East Africa, and India. Although it is estimated that 50,000–90,000 new cases occur globally each year, only 25–45% of these are officially reported, highlighting significant gaps in disease surveillance.^5^

In the absence of an effective human vaccine, disease treatment primarily relies on a limited pharmacological arsenal, including amphotericin B, miltefosine, and pentavalent antimonials. However, these therapeutic options come with significant drawbacks, such as high costs, complex long-term treatment protocols, and severe adverse effects, including nephrotoxicity, cardiotoxicity, and hepatotoxicity. The clinical utility of these agents is further constrained by emerging drug resistance and the requirement for parenteral administration in resource-limited settings. Consequently, there is a pressing need for innovative drug discovery approaches to expand the therapeutic landscape for this neglected tropical disease.^6–8^

Identifying antileishmanial hits poses a significant challenge in early stage drug discovery, particularly because of the time and resources required for experimental screening to assess efficacy and safety. Over recent decades, artificial intelligence (AI)-driven methodologies have revolutionized this process by significantly enhancing both the efficiency and cost-effectiveness of molecular property predictions.^9,10^ One of the core challenges in AI-based drug discovery is accurately representing chemical structures. Earlier machine learning approaches relied on simplistic representations, such as handcrafted features like molecular descriptors and fingerprints.^11–13^ Molecular descriptors quantify the physical and chemical attributes of compounds,^14^ while fingerprints encode the presence or absence of specific molecular substructures as bit vectors.^15^ Although these traditional methods have contributed significantly to previous efforts, they are inherently limited in their capacity to capture the nuanced structural and topological complexity of molecules, thus highlighting the need for more advanced and expressive representations in contemporary AI-driven drug discovery.^16,17^

Graph Neural Networks (GNNs) significantly advance this area, offering highly adaptable architectures and feature representations for modeling complex biological properties, such as antileishmanial activity.^18,19^ Unlike traditional deep learning models that represent molecules as linear sequences (e.g., SMILES strings) or bit vectors (e.g., molecular fingerprints), GNNs operate directly on molecular graphs, where atoms are represented as nodes and chemical bonds as edges. Formally, a molecular graph is defined as *G* = (*V*, *E*), with *V* denoting the set of atoms and *E* the set of bonds. Node features, such as atom types and hybridization states, are encoded in a feature matrix *X*, where each node *v* ∈ *V* is associated with an initial vector $x_{v}\in R^{d}$ . Bond attributes are similarly represented in an edge feature matrix. The connectivity of the molecule is captured by an adjacency matrix *A*, where *A*_*ij*_ = 1 indicates a bond between atoms *v*_*i*_ and *v*_*j*_.^20^

A wide range of GNN architectures, such as Message Passing Neural Networks (MPNN),^21^ Graph Attention Networks (GAT),^22^ Graph Isomorphism Networks (GIN),^23^ and Attentive Fingerprint (AttentiveFP),^24^ have been proposed and continually refined through the integration of sophisticated mechanisms. These include attention schemes,^25,26^ structure-aware update rules,^27,28^ hierarchical representation learning,^29,30^ and high-order relational modeling.^31^ In general, GNNs operate through a message-passing paradigm, wherein node features are iteratively updated by aggregating information from their immediate neighbors and, optionally, from edge features, using learnable functions that capture local chemical environments.^19^ This process facilitates the incorporation of both local and, to some extent, global structural context, ultimately enhancing the encoding of structural and contextual information and resulting in improved performance across diverse cheminformatics tasks.

Despite substantial progress in molecular representation learning, key methodological gaps remain in the field. Conventional message-passing architectures operate predominantly on local neighborhoods, which limits their ability to model long-range dependencies essential for capturing global pharmacophoric interactions.^32,33^ As network depth increases, these models are also susceptible to oversmoothing (node embeddings becoming increasingly indistinguishable) and oversquashing (compression of information from distant nodes through limited-capacity bottlenecks), which can reduce representational expressivity and degrade chemically meaningful signals.^34–36^

This study addresses these limitations by implementing a GNN-based framework that integrates well-established graph-learning mechanisms within the message-passing pipeline. We hypothesized that combining chemically grounded molecular representations with enhanced information propagation would improve activity prediction and support the prioritization of novel antileishmanial compounds. Using curated bioassay data from ChEMBL,^37^ proposed models demonstrated significant improvements in predictive performance over standard GNN counterparts in ablation studies. The framework also includes a counterfactual explainability module that highlights key substructures responsible for antileishmanial activity. The best-performing GNN models were subsequently employed in a large-scale virtual screening (VS) of 1.3 million commercially available compounds from the ChemBridge database, yielding 18 candidates for biological evaluation. This campaign achieved a 50% experimental hit rate and identified LC-61, a chemically novel scaffold exhibiting nanomolar antileishmanial potency, low cytotoxicity, and a favorable *in vitro* ADME profile, establishing it as a promising hit-to-lead candidate.

## 2 Results and Discussion

### 2.1 Data Description

A total of 1,415 compounds with reported *in vitro* antileishmanial activity against intracellular *L. infantum* were compiled from ChEMBL to support the development and evaluation of GNN models. These compounds were subsequently classified as active or inactive using two distinct activity cutoffs. A stringent 1 μM activity cutoff was applied to capture higher-potency profiles, resulting in 213 actives (IC_50_ ≤ 1 μM) and 1,220 inactives (IC_50_ > 1 μM), yielding a highly imbalanced data set with a lower prevalence of actives (Figure 1a). Additionally, a 10 μM activity cutoff, consistent with the hit criteria proposed by Katsuno et al.,^38^ was applied to generate a more balanced data set comprising 641 actives (IC_50_ ≤ 10 μM) and 774 inactives (IC_50_ > 10 μM) (Figure 1b).

**Figure 1:**
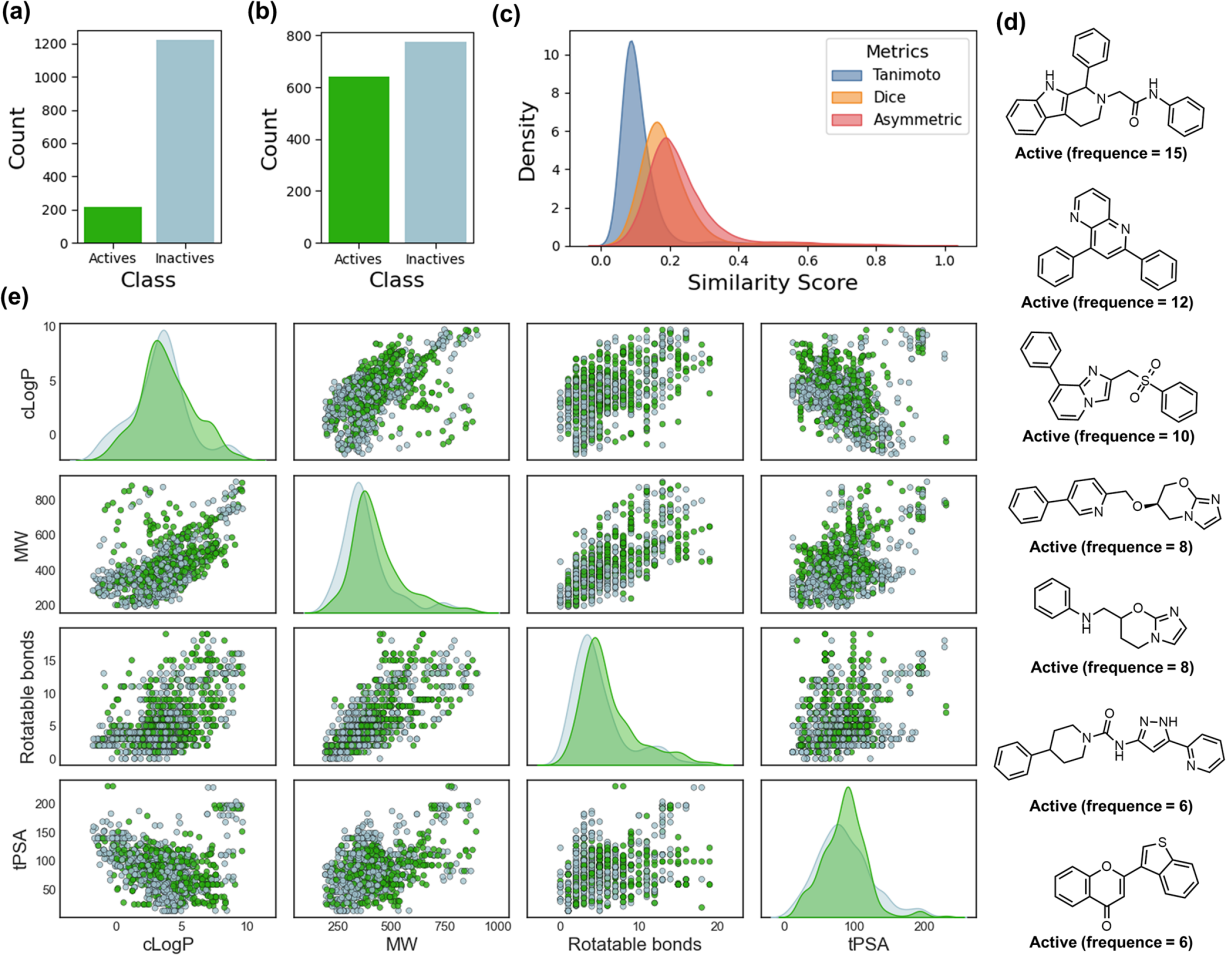
Chemical space analysis and structural profiling of active and inactive compounds used for model training. Distribution of actives and inactives in (a) imbalanced and (b) balanced data sets developed using 1 μM and 10 μM activity cutoffs, respectively. (c) Density plots of pairwise similarity scores between compounds calculated using Tanimoto, Dice, and Asymmetric metrics. (d) Molecular structures of representative scaffolds observed among actives, annotated with their frequency of occurrence in the balanced data set. (e) Pairwise scatter plots and kernel density estimates for cLogP, MW, number of rotatable bonds, and tPSA for actives (green) and inactives (gray) from the balanced data set.

Subsequently, pairwise molecular similarity distributions were computed using ECFP4 fingerprints with Tanimoto, Dice, and Asymmetric metrics, revealing overall low similarity scores across compound pairs and indicating a chemically diverse data set (Figure 1c). This diversity is essential for minimizing data set bias and enhancing scaffold-hopping capabilities in predictive pipelines. Furthermore, analysis of Bemis–Murcko scaffolds identified 659 distinct chemotypes across the data set. Among these, seven privileged scaffolds, frequently observed in actives but rarely in inactives, are shown in Figure 1d. These scaffolds predominantly comprise polyaromatic and heterocyclic frameworks, which are commonly enriched in bioactive libraries, suggesting the presence of privileged structures that may drive antileishmanial activity.

Scatter plot matrices and marginal density plots of key molecular descriptors, including calculated logarithm of the partition coefficient (cLogP), molecular weight (MW), number of rotatable bonds, and topological polar surface area (tPSA), were generated to visualize the physicochemical property distribution of active and inactive compounds. As shown in Figure 1e, substantial overlap was observed between actives and inactives across these physicochemical dimensions, underscoring the limitations of conventional property-based features for distinguishing antileishmanial activity in this data set. These findings highlight the need for advanced representation-learning approaches that capture nonlinear structure–activity relationships and topological patterns beyond classical descriptor-based methods.

### 2.2 Model Performance

To address the limitations of default message-passing GNNs, we implemented systematic engineering refinements in three established architectures (MPNN, GAT, and GIN), incorporating virtual nodes, skip connections, and jumping knowledge (JK) to enable multiscale molecular representation (Figure 2a).

**Figure 2:**
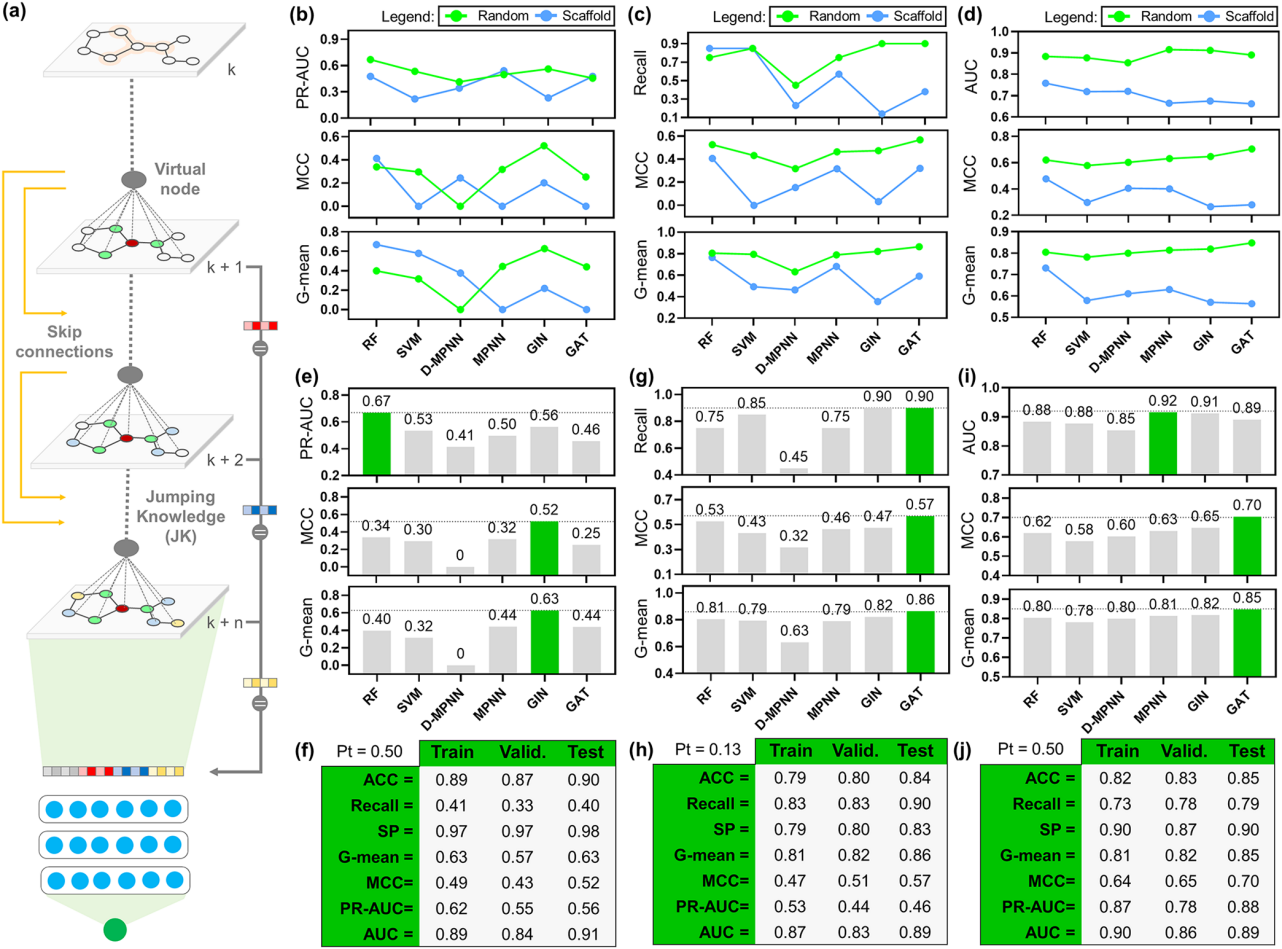
GNN engineering refinements and comparative performance across split strategies and class-imbalance regimes. (a) Schematic overview of the refined GNN design, integrating a virtual node, skip connections, and JK to support multiscale message aggregation. Test set performances under random and scaffold splits for the imbalanced data set (b) before and (c) after threshold-moving calibration, and (d) for the balanced data set. (e) Test-set performance of uncalibrated models on the imbalanced data set under the random split strategy. (f) Detailed test-set metrics of the best uncalibrated model (GIN) on the imbalanced data set. (g) Test-set performance of calibrated models on the imbalanced data set under the random split strategy. (h) Detailed test-set metrics of the best calibrated model (GAT) on the imbalanced data set. (i) Test-set performance of models on the balanced data set under the random split strategy. (j) Detailed test-set metrics of the best-performing model (GAT) on the balanced data set. Pt: predicted probability threshold.

Skip connections were incorporated to mitigate oversmoothing and gradient degradation during deep message-passing, preserving atom-level identity and pharmacophoric signals while ensuring stable training.^39,40^ In parallel, a virtual node was incorporated into each molecular graph to enrich chemical expressivity by forging long-range connections among pharmacophoric and structural features, thereby unifying local interactions within a global molecular context.^41^ This strategy strengthens the model’s ability to capture global information; however, it does not fully resolve the challenges arising when relevant signals are distributed across different topological ranges. In view of this, the JK mechanism was incorporated into GNN architectures to address this limitation by allowing the model to adaptively aggregate information across multiple message-passing depths, effectively capturing signals at varying neighborhood ranges.^42^

Subsequently, we benchmarked the refined GNNs against random forests (RFs), support vector machines (SVMs), and directed message-passing neural networks (D-MPNNs)^43,44^ under both random and scaffold-splitting regimes. Detailed statistical performance metrics for all generated models are provided in Tables S1–S3. As shown in Figure 2b, models trained using a random split consistently outperformed those trained using a scaffold split in the imbalanced data set (1 μM activity cutoff), with +14% in area under the precision-recall curve (PR-AUC), and +14.67% in Matthew’s correlation coefficient (MCC). To mitigate majority-class decision bias under class imbalance, we applied a threshold-moving calibration strategy,^45^ replacing the default 0.5 cutoff with a decision boundary empirically selected to maximize the G-mean based on the distribution of predicted probabilities. Following threshold-moving calibration (Figure 2c), the random-split models remained consistently superior to scaffold-split counterparts across all evaluated metrics.

We next evaluated model performance under the balanced setting (10 μM activity cutoff; Figure 2d). Under this regime, random-split training remained superior across all trained models, yielding gains of +19% in the area under the receiver operating characteristic curve (AUC) and +27.33% in MCC relative to scaffold-split counterparts.

Focusing on the best-performing split strategy, we then compared all models under the 1 μM activity cutoff. Before calibration, performance ranged from 0.41–0.67 for PR-AUC, 0.00–0.52 for MCC, and 0.00–0.63 for G-mean (Figure 2e). Curiously, RF achieved the highest PR-AUC (0.67), whereas GIN showed the best overall precalibration profile, with accuracy (ACC) = 0.90, G-mean = 0.63, and MCC = 0.52 (see Figure 2e,f). After threshold-moving calibration, G-mean increased substantially (∼41,16%), reaching 0.63–0.86 across models (Figure 2g). In contrast, PR-AUC and AUC remained unchanged, as threshold-moving only adjusts the decision threshold and does not alter the ranking of predicted probabilities. In this calibrated setting, the GAT model showed the best overall performance (Figure 2g,h), with ACC = 0.84, recall = 0.90, specificity (SP) = 0.83, G-mean = 0.86, and MCC = 0.57. Notably, in this setting, GAT outperformed the standard-gold D-MPNN on MCC (−25%) and G-mean (−23%), indicating improved class-balanced discrimination after threshold-moving calibration.

Under the balanced data set condition (10 μM activity cutoff; Figure 2i), discrimination performance remained high across all architectures, with AUC values ranging from 0.85 to 0.92. Although MPNN achieved the highest AUC (0.92), GAT again showed the best overall performance (Figure 2i,j), with test ACC = 0.85, MCC = 0.70, and G-mean = 0.85. Notably, GAT showed comparable-to-superior performance (+10% MCC, +5% G-mean) relative to the D-MPNN baseline across key classification metrics. These findings support GAT as the most robust and well-balanced method for predicting antileishmanial activity. However, additional performance may be achieved by integrating transformer-powered graph representation learning^46^ and link-based attributed graph clustering (approximate generative Bayesian learning)^47^ into the current message-passing pipeline, potentially improving representation expressiveness and scaffold-level generalization.

To complement this quantitative benchmarking, we next provide a qualitative view of how the selected GAT models organize their latent space using t-distributed stochastic neighbor embedding (t-SNE) projections. These visual patterns provide an intuitive, epoch-by-epoch depiction of how antileishmanial-relevant embeddings are organized during training. As shown in Figure 3a (imbalanced data set), the early projections (1st and fourth epochs) display a largely mixed organization, with active and inactive embeddings broadly overlapping, and the kernel-density curves along the first t-SNE dimension showing substantial overlap. By the 20th epoch, the embeddings reorganize into a more coherent manifold, and a class-related pattern begins to emerge, with actives concentrating more consistently within specific regions of the projection. This trend becomes more apparent by the 30th epoch, where active-enriched regions are more visually distinct, and the density profiles show reduced overlap. By the 42nd epoch, the projection indicates clearer spatial segregation of active- versus inactive-enriched regions, with more distinct density peaks. In Figure 3b (balanced data set), early epochs (1st and 10th) also show mixed organization, but class-related structuring becomes visually clearer by the 20th epoch, where active and inactive embeddings begin to occupy more distinct trajectories in the projection. The separation appears more pronounced by the 30th epoch. It remains evident at the 38th epoch, with active and inactive embeddings occupying largely distinct regions and the kernel-density plots showing more separated peaks along the first t-SNE dimension.

**Figure 3:**
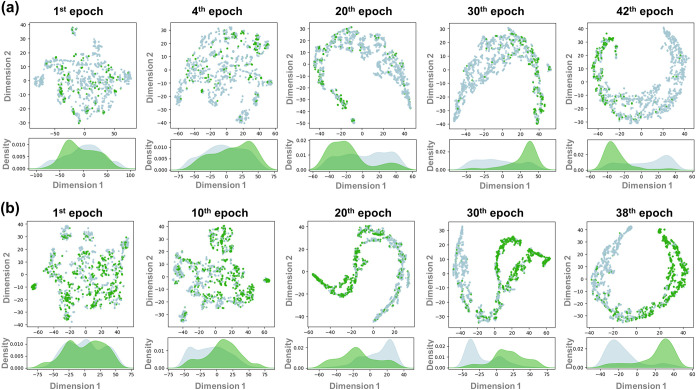
Temporal evolution of latent-space organization during training of GAT. (a) t-SNE projections for model trained on the imbalanced data set; (b) t-SNE projections for model trained on the balanced data set (selected epochs). Green and gray points represent embeddings of active and inactive compounds, respectively. Kernel-density plots of the first t-SNE dimension illustrate the evolving separation between active and inactive embeddings.

### 2.3 Ablation Study

A complementary ablation study (Figure 3) was conducted to evaluate the individual and combined contributions of multiscale components to model performance. Each component (virtual nodes, skip connections, and JK) was sequentially removed from the GAT architecture, and performance was evaluated on both antileishmanial data sets.

As shown in Figure 4a (imbalanced data set), the full GAT model (virtual node + skip connections + JK) achieved the highest PR-AUC = 0.46. Removing the virtual node produced only a minor decrease (0.46 → 0.44; −4.3%), whereas removing skip connections caused the largest single-component drop (0.46 → 0.35; −23.9%). Ablating JK led to a modest reduction (0.46 → 0.43; −6.5%). For combined removals, PR-AUC decreased to 0.40 when removing virtual node + skip (−13.0%), to 0.41 when removing virtual node + JK (−10.9%), and to 0.36 when removing skip + JK (−21.7%). The complete removal of all three mechanisms yielded the lowest performance (0.46 → 0.32; −30.4%). A parallel pattern emerged under balanced conditions. The full GAT model also achieved the best discrimination (AUC = 0.89) in the balanced data set (Figure 3b). Removing the virtual node resulted in a small decrease (0.89 → 0.86; −3.4%), whereas removing skip connections led to a moderate drop (0.89 → 0.82; −7.9%). In contrast, removing JK caused a marked drop (0.89 → 0.69; −22.5%). For combined ablations, AUC decreased to 0.74 when removing the virtual node + skip connections (−16.9%) and to 0.75 when removing the virtual node + JK (−15.7%). The joint removal of skip + JK led to an AUC of 0.79 (−11.2%). Finally, removing all three components yielded the lowest AUC (0.69; −22.5%). Overall, skip connections accounted for the largest PR-AUC gains in the imbalanced data set, whereas JK produced the largest AUC drop in the balanced data set, with the virtual node showing a consistently smaller effect across both settings.

**Figure 4:**
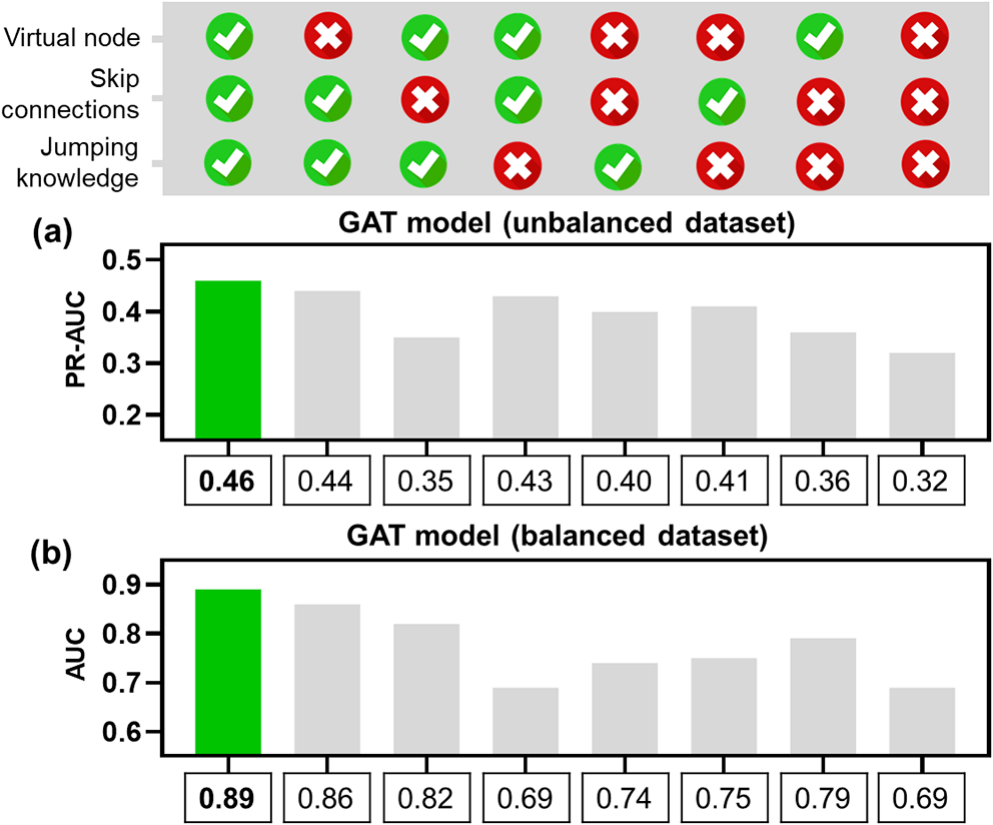
Ablation study of multiscale components in GAT models for antileishmanial activity prediction. (a) denotes PR-AUC values for GAT models trained on the imbalanced data set and (b) denotes AUC values for GAT models trained on the balanced data set, evaluated across different combinations of virtual node, skip connections and JK components.

### 2.4 Model Explainability

To elucidate the structural drivers of the GAT predictions, we performed a counterfactual perturbation analysis on the model trained under the balanced data set. Contribution maps were generated by systematically perturbing local functional-group, molecular-bond, or ring environments via chemically valid isosteric replacements, and the resulting prediction shifts were quantified relative to the unperturbed reference prediction. The resulting maps provide a direct, model-agnostic estimate of how specific substructures influence predicted antileishmanial activity. Methodologically, this approach is consistent with recent advances in molecular counterfactual explainability, including the SELFIES-based framework of Wellawatte et al.,^48^ for generating chemically valid molecular-level counterfactuals.

As shown in Figure 5a–i (actives), our counterfactual approach highlighted chemically intuitive activity-supporting regions (red) that concentrate around nitro-aromatic groups, fluorine-containing substituents, carbonyl-containing linkers, sulfonamide-like functionalities, nitriles, and protonatable nitrogens. Across these active scaffolds, red hotspots frequently localize at key hydrogen-bond donor/acceptor centers and along heteroatom-bearing connectors that bridge aromatic substructures and five-member heteroaromatic rings (imidazole and pyrazole). In contrast, negative contributions (blue) in the active set tend to appear on peripheral aromatic rings and chlorine-containing substituents. In Figure 5j–o (inactives), the maps exhibit an inverted or attenuated pattern relative to the actives. Blue regions are more prominent and often span large portions of the molecular core or extended aromatic systems and six-member aliphatic rings, indicating that perturbations in these areas consistently move predictions further toward inactivity. However, these attribution patterns should be interpreted cautiously. The apparent contribution of a motif can vary across chemotypes, as differences in connectivity, substituent context, and overall physicochemical properties can alter how a substructure influences the model’s prediction.

**Figure 5:**
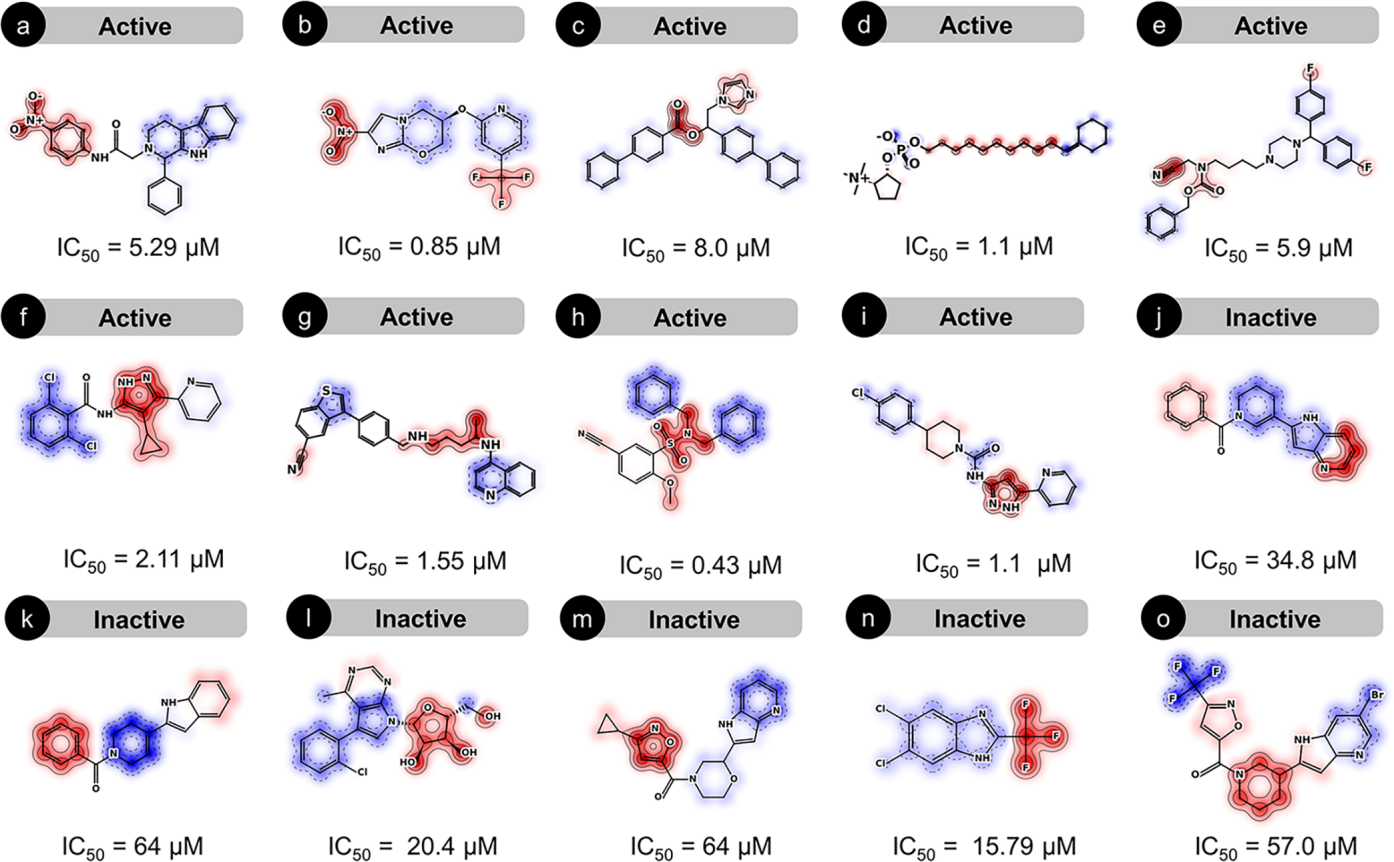
Counterfactual explainability of predicted antileishmanial activity. (a–i) shows counterfactual maps for selected actives while (j–o) shows counterfactual maps for selected inactives. Red contours indicate regions contributing positively to antileishmanial activity, whereas blue contours indicate regions contributing negatively.

To further contextualize these findings, we additionally inspected attention-weight visualizations for the same set of molecules (Supporting Figure S1) as an alternative strategy for model interpretability. However, these visualizations lacked clear patterns linking specific molecular substructures to predicted activity, limiting their practical interpretability in this setting. This limitation is consistent with recent reports questioning whether attention weights in GNNs reliably reflect feature importance.^49^ Accordingly, it highlights the advantage of our counterfactual perturbation-based framework, which provides more direct and chemically grounded contribution estimates.

### 2.5 Virtual Screening

A virtual screening workflow was designed to prioritize novel antileishmanial candidates from a commercially available library of 1.3 million compounds (Figure 6). Initially, the GAT model trained with a 10 μM activity cutoff was used as a primary filter, identifying approximately 20,000 compounds predicted to be active with high confidence. Subsequently, the GAT model trained on the more stringent 1 μM activity cutoff was used as a secondary filter to refine the data set, yielding a subset of 5,000 compounds for downstream evaluation. To ensure the drug-likeness and favorable pharmacokinetic properties, a physicochemical filter was applied based on aqueous solubility (cLogS > −5.0) and lipophilicity (cLogP between 0.5 and 4.0), narrowing the compound set to 1,000 structures. Finally, a structural novelty filter was implemented using Tanimoto similarity with ECFP4 fingerprints to exclude highly similar compounds to known antileishmanial agents, ensuring the selection of structurally novel candidates. The nearest neighbors identified for these putative hits are provided in Table S4 for reference. Final selection incorporated medicinal chemistry expertise through visual inspection of the molecular structures to remove candidates with undesirable features. This multitiered screening cascade identified 18 putative hits, representing chemically diverse compounds suitable for further *in vitro* evaluation against *L. infantum*.

**Figure 6:**
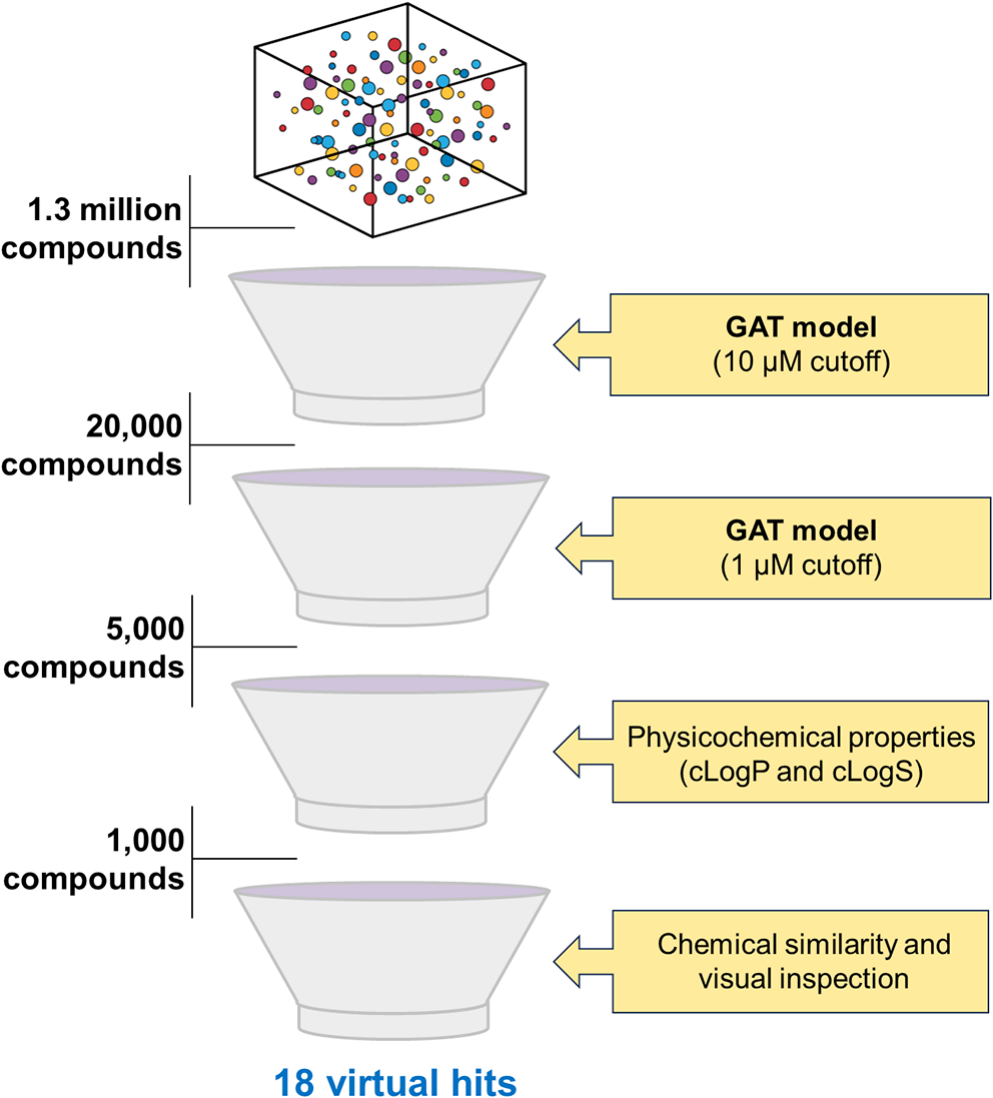
Virtual screening workflow for identifying novel antileishmanial compounds. The physicochemical filter was based on aqueous solubility (cLogS > −5.0) and lipophilicity (cLogP between 0.5 and 4.0). A structural novelty filter was applied using Tanimoto similarity with ECFP4 fingerprints to ensure that the putative hits had no prior experimental activity reported against *Leishmania* spp.

### 2.6 Experimental Validation

The 18 putative hits were evaluated *in vitro* against promastigotes of *L. infantum* (Table 1). As a result, 11 compounds exhibited significant antileishmanial activity, with IC_50_ values ranging from 0.037 to 11.7 μM. To further characterize the prioritized hits, intracellular assays were performed in THP-1-derived macrophages infected with *L. infantum*, followed by cytotoxicity assessments to determine selectivity index (SI = CC_50_/IC_50_). Following published guidance for antileishmanial hit identification,^38^ compounds were considered experimentally validated hits if they inhibited intracellular IC_50_ < 10 μM and showed an acceptable host-cell SI > 10.

**Table 1:**
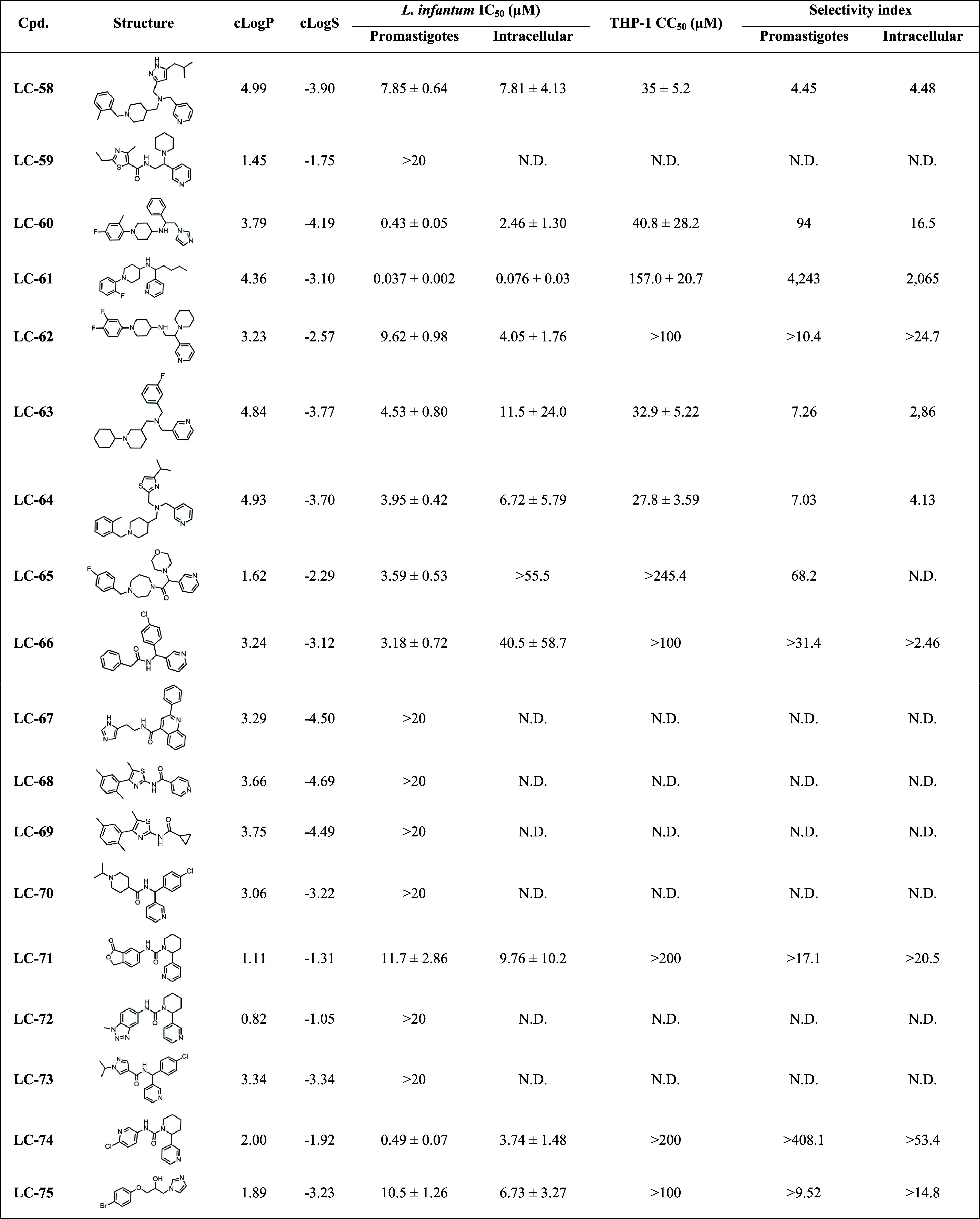
*In Vitro* Antileishmanial Activity and Selectivity of Prioritized Compounds against *L. infantum*

As shown in Table 1, eight compounds maintained activity in the intracellular assay, with IC_50_ values ranging from 0.076 to 11.5 μM, underscoring the robustness of the screening cascade and the predictive power of our GNN framework. Four compounds (LC-58, LC-63, LC-64, and LC-66) exhibiting low intracellular potency (IC_50_ > 10 μM) and/or insufficient selectivity index (SI < 10) were deprioritized due to their limited potential for hit-to-lead optimization.^38^ In contrast, six compounds (LC-60, LC-61, LC-62, LC-71, LC-74, and LC-75) demonstrated intracellular IC_50_ values <10 μM while maintaining SIs > 17.

Among the validated hits, LC-60, LC-61, and LC-74 emerged as the top three candidates. LC-60 displayed nanomolar potency against promastigotes (IC_50_ = 0.43 ± 0.05 μM) and submicromolar potency against intracellular amastigotes (IC_50_ = 2.46 ± 1.30 μM), while exhibiting low cytotoxicity in THP-1 cells (CC_50_ = 40.8 ± 28.2 μM, SI > 16.5). LC-74 likewise achieved nanomolar potency against promastigotes (IC_50_ = 0.49 ± 0.07 μM) and submicromolar potency against intracellular amastigotes (IC_50_ = 3.74 ± 1.48 μM), while exhibiting low THP-1 cytotoxicity (CC_50_ > 200 μM, SI > 53.4). Notably, LC-61 emerged as the most promising hit, exhibiting potent nanomolar activity against both promastigotes (IC_50_ = 0.037 ± 0.002 μM) and intracellular amastigotes (IC_50_ = 0.076 ± 0.03 μM), combined with minimal cytotoxicity in THP-1 cells (CC_50_ = 157 μM) and SI > 2,000. Benchmarking against clinical comparators highlights LC-61′s advantage. Amphotericin B shows comparable potency against intracellular *L. infantum* amastigotes (IC_50_ = 0.14 μM) but exhibits high cytotoxicity (CC_50_ = 1.2 μM), yielding a SI = 8.6.^50^ Similarly, miltefosine demonstrated lower potency (IC_50_ = 0.37 μM) and moderate cytotoxicity (CC_50_ = 51.1 μM, SI = 138).^50^

### 2.7 Early ADME Profiling of Hits

To prioritize candidates for hit-to-lead optimization, *in vitro* ADME profiles were determined for compounds LC-60, LC-61, and LC-74. Comprehensive experimental details and analytical data are provided in the Supporting Information (Files S1, S2, S3, S4, and S5). As shown in Table 2, all three hits showed high aqueous solubility at acidic and physiological pHs (≈28–33 μg/mL). At pH 2.0, LC-60 and LC-61 exceeded 28 μg/mL (>32.21 and >28.35 μg/mL, respectively), while LC-74 reached 27.58 μg/mL; at pH 7.4, LC-74 was the most soluble (32.81 μg/mL), followed by LC-60 (>30.03 μg/mL) and LC-61 (>28.18 μg/mL). Experimentally measured distribution coefficients (eLogD) were within a drug-like range: LC-60 and LC-61 showed values near the Lipinski Rule-of-Five cutoff (4.45 and 4.07, respectively), whereas LC-74 was less lipophilic (2.39). Consistent with the observed eLogD values, parallel artificial membrane permeability assays (PAMPA) showed high apparent passive permeability for all three compounds (4.86–10.85 × 10^–6^ cm/s): LC-74 was highest (10.85 × 10^–6^ cm/s), followed by LC-60 (6.36 × 10^–6^ cm/s) and LC-61 (4.86 × 10^–6^ cm/s).

**Table 2:** Summary of *In Vitro* ADME Properties for Top Compounds

			kinetic solubility		HLM		MLM	
Cpd.	eLogD	PAMPA (10^–6^ cm/s)	pH 2.0 (μg/mL)	pH 7.4 (μg/mL)	CL_int_ (μL/min/mg)	*T*_1/2_ (min)	CL_int_ (μL/min/mg)	*T*_1/2_ (min)
**LC-60**	4.45	6.36	>32.21	>30.03	54.80	50.59	119.6	23.18
**LC-61**	4.07	4.86	>28.35	>28.18	259.6	10.68	242.8	11.42
**LC-74**	2.39	10.85	27.58	32.81	18.00	154.0	27.20	101.93

Microsomal stability assays revealed distinct metabolic profiles among the three hits (Table 2). LC-60 exhibited moderate intrinsic clearance in human liver microsomes (HLM, CL_int_ = 54.80 μL/min/mg) with shorter half-life (*T*_1/2_ = 50.59 min), while clearance in mouse liver microsomes (MLM) was lower (CL_int_ = 119.6 μL/min/mg; *T*_1/2_ = 23.18 min), suggesting species-dependent turnover. LC-74 showed the highest microsomal stability across both systems, with low clearance values (MLM CL_int_ = 27.20 μL/min/mg; HLM CL_int_ = 18.0 μL/min/mg) and adequate half-lives (101.93 and 154.0 min, respectively), consistent with its lower lipophilicity (eLogD = 2.39). In contrast, LC-61 exhibited a pronounced metabolic turnover, characterized by higher intrinsic clearance in both species (MLM CL_int_ = 242.8 μL/min/mg; HLM CL_int_ = 259.6 μL/min/mg) and correspondingly short half-lives (MLM *T*_1/2_ = 11.42 min; HLM *T*_1/2_ = 10.68 min). Notably, the elevated clearance of LC-61 occurred despite its balanced lipophilicity (eLogD = 4.07), suggesting that rapid turnover stems from structural liabilities rather than global physicochemical properties.

The presence of an unprotected aromatic ring likely renders the molecule vulnerable to hydroxylation catalyzed by cytochrome P450 isoforms, consistent with the higher clearance observed in human microsomes. Such metabolic instability could be mitigated through targeted electronic modulation (e.g., selective fluorination or methoxylation) to shield the reactive aromatic site while preserving potency and physicochemical balance. Despite its moderate metabolic liability, LC-61 remains the most promising hit, combining nanomolar potency, favorable permeability, and an overall drug-like profile that strongly supports its advancement into hit-to-lead optimization. In addition, LC-61 demonstrates favorable synthetic accessibility, with a SwissADME^51^ Synthetic Accessibility Score (SAscore) of 3.18, indicating that the compound can be obtained through feasible synthetic routes and supporting its potential for further lead optimization.

### 2.8 Target Fishing

To elucidate the potential mechanism of action of LC-61, a target fishing analysis was implemented through a multistep *in silico* approach. Initially, the pyridine moiety of LC-61 was used to conduct a substructure search in the ChEMBL database and the scientific literature. This approach supported the identification of potential targets associated with this moiety. Based on this rationale, *L. infantum* sterol C4-methyl oxidase (CYP5122A1)^52,53^ and sterol 14α-demethylase (CYP51)^54,55^ were prioritized for further evaluation due to their documented tractability as targets for pyridine-based inhibitors. These cytochrome P450 enzymes catalyze sequential oxidative steps in the ergosterol biosynthesis pathway in *Leishmania*, with CYP5122A1 forming C4-oxidation metabolites and CYP51 mediating sterol 14α-demethylation.^52,53^

Subsequently, molecular docking simulations were performed against the AlphaFold-predicted structure of *L. infantum* CYP5122A1 (Figure 7a) and the crystal structure of *L. infantum* CYP51 ^56^ (Figure 7b). However, initial analysis of docking scores (GlideScore function) using a benchmark data set of known actives and decoys yielded poor enrichment performance, with CYP5122A1 achieving an AUC of 0.54 and both Boltzmann-Enhanced Discrimination of ROC (BEDROC_1%_) and Enrichment Factor (EF_1%_) values of 0.0 (Figure 7c). At the same time, CYP51 showed an AUC of 0.69 with BEDROC_1%_ and EF_1%_ similarly at 0.0 (Figure 7d). These outcomes demonstrate that GlideScore failed to discriminate actives from decoys for either target, with AUCs near random expectation and no early enrichment, thereby lacking target-fishing utility for reliable target assignment.

**Figure 7:**
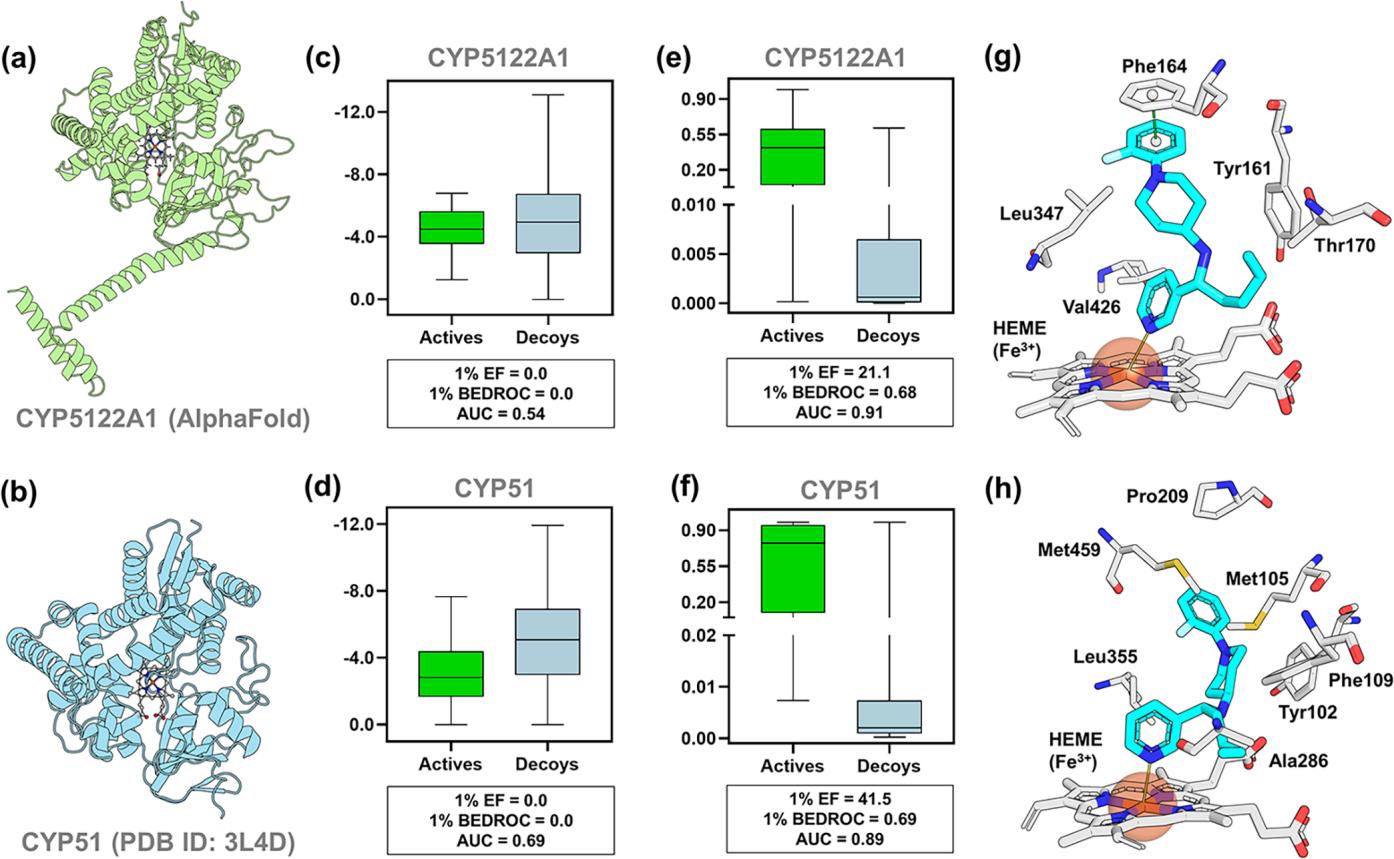
Target fishing and molecular docking of LC-61. (a) Predicted 3D structure of *L. infantum* CYP5122A1 and (b) X-ray structure of *L. infantum* CYP51 (PDB ID: 3L4D), respectively. Docking scores corresponding enrichment metrics for (c) CYP5122A1 and (d) CYP51 obtained using a benchmark data set of known actives and decoys. Bayesian rescoring and corresponding enrichment metrics for (e) CYP5122A1 and (f) CYP51 obtained using a benchmark data set of known actives and decoys. Predicted binding poses of LC-61 in the active sites of (g) CYP5122A1 and (h) CYP51, highlighting key interactions with active site residues and HEME.

To enhance the reliability of the docking protocol, a Bayesian rescoring function was developed using raw energy terms and scoring descriptors extracted from the docking outputs. This approach substantially improved predictive performance across all metrics, with CYP5122A1 reaching an AUC of 0.91, BEDROC_1%_ of 0.68, and EF_1%_ of 21.1 (Figure 7e), and CYP51 achieving an AUC of 0.89, BEDROC_1%_ of 0.69, and EF_1%_ of 41.5 (Figure 7f). While initial docking yielded limited enrichment, Bayesian rescoring significantly improved discrimination, underscoring the limitations of raw docking scores for novel scaffolds. These results confirm the effectiveness of the Bayesian rescoring strategy in enhancing the docking protocol’s ability to distinguish actives from decoys.

Following validation of the docking protocol, LC-61 emerged as the only candidate predicted to inhibit both CYP5122A1 and CYP51. In CYP5122A1 (Figure 7g), LC-61 adopted a stable binding pose within the active site, with the fluorobenzene ring forming a π–π stacking interaction with Phe164, while the butyl substituent engaged in hydrophobic contacts with Tyr161 and Thr170, contributing to the stabilization of the ligand within the binding pocket. Notably, the pyridine nitrogen was oriented for axial coordination with HEME iron, a key interaction commonly exploited by CYP inhibitors.

In CYP51 (Figure 7h), LC-61 also displayed a favorable binding orientation, with fluorobenzene engaging hydrophobic contacts with Met105, Pro209, and Met459. In addition, the pyridine nitrogen was positioned for axial coordination with the HEME iron, mirroring the key interaction observed in CYP5122A1, while the butyl substituent engaged in hydrophobic contacts with Ala286, Phe109, and Tyr102. These findings are consistent with recent work demonstrating that dual inhibition of CYP5122A1 and CYP51 is required for optimal antileishmanial activity, whereas inhibition of CYP51 alone yields limited efficacy due to compensatory pathways in ergosterol biosynthesis.^57^ Notably, LC-61 was the most potent compound identified in this study, supporting the notion that concurrent blockade of C4-methyl oxidation and C14-demethylation enhances antileishmanial efficacy. Importantly, the scaffold of LC-61 offers multiple opportunities for derivatization, which could enable fine-tuning of potency and metabolic stability.

## 3 Conclusions

In this study, GATs achieved strong predictive performance for antileishmanial activity. Complementing these predictions, counterfactual explainability based on isosteric perturbations localized group- and ring-level drivers and enabled rational prioritization of activity-related structural motifs. The application of this framework to a chemically diverse library of 1.3 million compounds yielded 18 putative hits, with a 50% hit rate in *in vitro* experimental validation, underscoring its practical utility in accelerating antileishmanial hit discovery. Among these, LC-61 emerged as most promising hit, featuring a chemically novel scaffold with nanomolar potency against intracellular amastigotes (IC_50_ = 0.076 μM), low cytotoxicity (CC_50_ = 157 μM), and a highly favorable SI (>2000). Its balanced kinetic solubility (>28 μg/mL at pH 2.0–7.4), lipophilicity (eLogD = 4.07), and high passive permeability (PAMPA = 4.86 × 10–6 cm/s) further support its advancement despite moderate microsomal turnover, which can be mitigated by targeted electronic modifications to the aromatic ring. These findings highlight LC-61 as a validated and pharmacokinetically tractable hit for prospective hit-to-lead optimization and demonstrate the broader potential of our GNN framework to streamline the discovery of novel antileishmanial compounds.

## 4 Experimental Section

### 4.1 Data Collection and Curation

Compounds with IC_50_ data obtained after 72 h of exposure against intracellular amastigotes of *Leishmania infantum* (CHEMBL612848) were retrieved from the ChEMBL database.^37^ Subsequently, samples were labeled as active or inactive using activity cutoffs of 1 μM and 10 μM, providing clear class delineation for subsequent predictive modeling analyses. Chemical structures in simplified molecular-input line-entry system (SMILES) format and corresponding bioactivity data were meticulously curated following the guidelines proposed by Fourches et al.^58,59^ Curation steps included the normalization of nitro groups and aromatic systems, removal of salts, mixtures, polymers, and organometallic compounds, as well as standardization of tautomeric forms to ensure consistency across the data set. For duplicate entries with discordant activities, all corresponding records were excluded, while duplicates with concordant activities were consolidated to a single representative entry.

### 4.2 Data Description

Chemical diversity within the data set was assessed by calculating pairwise molecular similarity distributions using ECFP4 fingerprints with Tanimoto, Dice, and Asymmetric similarity metrics. Molecular fingerprints were generated using the RDKit framework v.2024.4.5, and similar distributions were visualized using kernel density estimates. In parallel, physicochemical profiles were characterized using cLogP, MW, number of rotatable bonds, and tPSA computed for each compound using RDKit descriptor functions. Subsequently, scatter plot matrices and marginal density plots were generated to visualize the distribution of these physicochemical descriptors across actives and inactives.

### 4.3 GNNs

#### 4.3.1 Split of Data Sets

The curated data sets were partitioned into training, validation, and test sets in an 8:1:1 proportion, using random and scaffold-based splitting schemes. In the random split, compounds were assigned to subsets via uniform sampling. For the scaffold split, compounds were grouped by Bemis–Murcko scaffolds, and each scaffold group was confined to a single subset, preventing data leakage and providing a more stringent assessment of generalization. The training set was used to fit the models, the validation set to tune hyperparameters, and the held-out test set to assess final performance.

#### 4.3.2 Feature Representation

Molecular graphs were generated from SMILES using RDKit, with atoms represented as nodes and bonds as edges. Atom-level features included one-hot encoding of atomic number, degree, formal charge, hybridization state, and aromaticity. Bond-level features were encoded using one-hot representations of bond type, conjugation status, ring membership, and stereochemistry, including chirality. Feature vectors were built as tensors to provide structured graph representations during model training.

#### 4.3.3 Architectural Refinements

First, skip connections enable the models to combine low-level and high-level features, facilitating better training dynamics and expressiveness as shown in eq 1

1 $$h^{\left(l\right)}\leftarrow h^{\left(l\right)}+h^{\left(l-1\right)}$$

Where $h^{\left(l-1\right)}$ , $h^{\left(l\right)}\in R^{d}$ are input and output features at layer l . This additive transform acts as identity initialization, preserving gradient flow. For dimension mismatches, linear projections align features before addition.

In parallel, a virtual node was incorporated. This node is initialized with a learnable embedding by $h_{\mathrm{virt}}^{\left(0\right)}\in R^{d}$ and interacts with all atomic nodes by contributing additively to their representations at each message-passing layer. For a given graph *G* = (*V*, *E*), the representation of each atom *v* ∈ *V* at layer l is modified as shown in eq 2

2 $$h_{v}^{\left(l\right)}\leftarrow h_{v}^{\left(l\right)}+h_{\mathrm{virt}}^{\left(l\right)}$$

where $h_{v}^{\left(l\right)}$ is the atom’s representation at layer l , and $h_{\mathrm{virt}}^{\left(l\right)}$ is the virtual node’s representation at the same layer. This additive interaction ensures that global molecular information is dynamically propagated to all atomic nodes. After message passing, the virtual node embedding is updated by aggregating the current node states across graph as shown in eq 3

3 $$h_{\mathrm{pool}}^{\left(l\right)}=\sum_{v\in V}h_{v}^{\left(l\right)}$$

where $h_{\mathrm{pool}}^{\left(l\right)}$ is the graph-level summary at layer l , computed as permutation-invariant sum over all atomic representations $h_{v}^{\left(l\right)}$ . The pooled representation is then transformed through a learnable nonlinear function ${\mathrm{MLP}}^{\left(l\right)}$ , typically consisting of two linear layers with intermediate activation and normalization, to produce a virtual node embedding $\Delta h_{\mathrm{virt}}^{\left(l+1\right)}$ (eq 4)­

4 $$\Delta h_{\mathrm{virt}}^{\left(l+1\right)}={\mathrm{MLP}}^{\left(l\right)}\left(h_{\mathrm{pool}}^{\left(l\right)}\right)$$

This ensures the virtual node serves as a contextual relay, iteratively refining global graph-level context while retaining memory of prior states. Finally, the virtual node embedding is updated by residual addition as shown in eq 5

5 $$h_{\mathrm{virt}}^{\left(l+1\right)}=h_{\mathrm{virt}}^{\left(l\right)}+\Delta h_{\mathrm{virt}}^{\left(l+1\right)}$$

Where the virtual node embedding from the previous layer $h_{\mathrm{virt}}^{\left(l\right)}\in R^{d}$ is incremented by the newly computed update $\Delta h_{\mathrm{virt}}^{\left(l+1\right)}\in R^{d}$ . This residual update scheme allows the virtual node to dynamically absorb and redistribute information, functioning as a central relay for integrating subgraph signals and contextualizing local features within a molecule-level representation.

For the Jumping Knowledge, let H^(0)^, H^(1)^, ..., H^(*L*)^ denote the sequence of node embeddings obtained at each of the *L* message-passing layers (eq 6). JK aggregation builds a unified node representation through feature concatenation

6 $$H^{\mathrm{JK}}=\mathrm{concat}\left[H^{\left(0\right)},H^{\left(1\right)},...,H^{\left(L\right)}\right]$$

This final embedding $H^{\mathrm{JK}}\in R^{\left|V\right|\times d_{\mathrm{JK}}}$ , where $d_{\mathrm{JK}}=\sum_{l=0}^{L}d^{\left(l\right)}$ captures multiscale structure features by aggregating both shallow and deep node contexts. Subsequently, a graph-level embedding $h_{G}\in R^{d_{\mathrm{JK}}}$ is obtained via pooling function over node embeddings as shown in eq 7

7 $$h_{G}=\mathrm{AGG}\left(\{h_{v}^{\mathrm{JK}}|v\in V\}\right)$$

Depending on the architecture and task requirements, AGG denotes a permutation-invariant function such as summation, averaging, or attention-weighted pooling. This strategy ensures that the model captures the hierarchical nature of chemical graphs, from local functional groups to extended rings and substructures, thereby enhancing its ability to generalize across complex molecules.

#### 4.3.4 Model Training and Evaluation

GNNs were implemented and trained using PyTorch v.2.5.1 and PyTorch Geometric v.2.6.1 on an NVIDIA TITAN Xp GPU. On-the-fly batches with sizes of 64 and 48 were used for the imbalanced and balanced data sets, respectively. Models were trained for a maximum of 2,000 epochs using binary cross-entropy (BCE) loss, and early stopping was applied to prevent overfitting. Training terminates when no improvement in validation loss is observed within the adaptive five-epoch window; otherwise, the best validation loss checkpoint is refreshed. Regularization strategies, including gradient clipping and learning rate scheduling (cosine annealing with warm-up), were employed to improve generalization. Pruning strategies were applied during optimization to terminate unpromising trials early. Hyperparameter optimization was conducted using Optuna v.4.2.0, with 50 trials per architecture to explore the search space systematically. Model performance was evaluated using ACC, recall, SP, MCC, and AUC, with G-mean and PR-AUC additionally considered to address the imbalanced nature of the imbalanced data set. Finally, embedding vectors were saved during training and projected for visualization using t-SNE and kernel density estimation (KDE) to monitor the organization of the latent space across epochs.

#### 4.3.5 Threshold-Moving

A post-training calibration procedure was implemented using a threshold-moving strategy^45^ to refine decision boundaries derived from raw predicted probabilities. Thresholds were evaluated individually for each model across a 0.0–1.0 range with 0.01 increments on the training and validation sets, and optimal values were defined by the highest G-mean as shown in eq 8

8 $$\tau^{*}=\underset{\tau \in \left\{0.00,0.01,...,1.00\right\}}{\arg\;\max}\sqrt{\mathrm{recall}\left(\tau \right)\cdot \mathrm{SP}\left(\tau \right)},,{ŷ}_{i}\left(\tau \right)=1\left[{\mathrm{p̂}}_{i}\geq \tau \right]$$

where $\sqrt{\mathrm{recall}\left(\tau \right)\cdot \mathrm{SP}\left(\tau \right)}$ denote G-mean computed at threshold τ. The indicator function 1­[*p̂*_*i*_ ≥ τ] assigns a binary label *y*_*^i*_(τ) to each predicted probability *p̂*_*i*_, with outcome 1 if the predicted probability is greater than or equal to τ, and 0 otherwise. The calibrated thresholds τ_t_* were subsequently applied to the test set to reclassify predictions, enhancing the trade-off between recall and SP while maintaining PR-AUC and AUC.

#### 4.3.6 Model Explainability

A counterfactual approach was developed to generate contribution maps from chemically valid graph perturbations. Initially, ∼910 functional-group, bond-level, or ring-level isosteric perturbations, organized into 18 rule families (see Table S5), were applied to the molecular graph, and the resulting prediction shifts were quantified relative to the unperturbed reference prediction. All transformations were executed under structural admissibility constraints to preserve chemical connectivity and validity. The prediction shifts for each valid perturbed candidate *k* were computed as shown in eq 9

9 $$\Delta^{\left(k\right)}=p_{0}-p_{i}^{\left(k\right)}$$

where *p*_0_ is the predicted probability for the unperturbed graph, *p*_*i*_^(*k*)^ is the predicted probability for the *k*th valid perturbed graph, and Δ^(*k*)^ is the corresponding prediction shift. If perturbation *k* is valid, this shift was equally distributed across all perturbed atoms, as defined in eq 10

10 $$\delta_{i}^{\left(k\right)}=\frac{\Delta^{\left(k\right)}}{\left|G^{\left(k\right)}\right|},i\in G^{\left(k\right)}$$

where δ_*i*_^(*k*)^ is the contribution assigned to atom *i* under perturbation *k*, *G* ^(*k*)^ is the set of affected atoms and bonds, and |*G* ^(*k*)^| is its cardinality. Then, atom-level contributions were pooled across assigned valid perturbations, and the mean local effect was estimated as shown in eq 11

11 $$\mu_{i}=\frac{1}{\left|P_{i}\right|}\sum_{k\in P_{i}\;}\delta_{i}^{\left(k\right)}$$

Where μ_*i*_ is the mean perturbation effect for atom *i*, *P*_*i*_ = {*k*: *i* ∈ *G* ^(*k*)^} is the set of valid perturbations affecting atom *i*, and |*P*_*i*_| is the number of valid contributions assigned to atom *i*. To capture directional consistency of perturbation effects while avoiding redundant positive/negative frequency terms, a signed agreement index was computed as shown in eq 12

12 $$\kappa_{i}=\frac{1}{\left|P_{i}\right|}\sum_{k\in P_{i}\;}{\mathrm{sgn}(\delta }_{i}^{\left(k\right)})$$

where κ_*i*_ corresponds to the difference between the empirical frequencies of positive and negative contributions for atom *i*, with κ_*i*_ ∈ [−1,1], and sgn­(*x*) = +1 if *x* > 0, sgn­(*x*) = 0 if *x* = 0, and sgn­(*x*) = −1 if *x* < 0. Finally, atom-level relevance was obtained by combining effect magnitude and directional agreement (eq 13)­

13 $$h_{i}=\mu_{i}\kappa_{i}$$

where *h*_*i*_ is the final importance score for atom *i*. Nonzero *h*_*i*_ values were normalized (zero mean, unit variance) and projected onto 2D chemical structures via RDKit’s similarity maps for visualization. Additionally, atom-level attention weights from attention-based GNNs were extracted, providing a complementary visualization of substructural relevance.

### 4.4 Benchmarking

To ensure a rigorous and fair methodological comparison, we benchmarked the refined GNN architectures against machine learning (RF and SVM) and deep learning (D-MPNN) baselines. RF and SVM were implemented in Scikit-learn v0.24.2 using ECFP4 fingerprints as input features, and their hyperparameters were optimized via Bayesian optimization in Scikit-Optimize v.0.7.4 under a 5-fold cross-validation scheme. The D-MPNN was implemented in Chemprop v.2.2.1 and trained using the same data split protocols adopted for the proposed models, to guarantee direct comparability across methods.

### 4.5 Target Fishing

#### 4.5.1 Substructure Search

Initially, a SMARTS-based substructure search was conducted in ChEMBL and the scientific literature using the LC-61 pyridine ring to retrieve bioactivity data for Leishmania proteins whose ligands contained this same ring. *L. infantum* target candidates were then limited to enzymes with reported roles in sterol biosynthesis, identifying CYP5122A1 and CYP51 for subsequent *in silico* evaluation.

#### 4.5.2 Protein Preparation

The AlphaFold2-predicted CYP5122A1 model (UniProt ID: A4I2K5) and the X-ray structure of CYP51 (PDB ID: 3L4D) were processed using Protein Preparation Wizard in the Maestro workspace v.9.3 (Schrödinger, LCC, New York, 2012). In this step, hydrogen atoms were added to the proteins, and bond orders and formal charges were adjusted accordingly. Further, the Epik^60^ was employed to predict the protonation states (p*K*_a_) of polar amino acids at pH = 7.4 ± 0.5, whereas the PROPKA v.3.1 ^61,62^ was used to optimize the hydrogen orientations. Subsequently, loop regions in the CYP5122A1 model were rebuilt using Prime’s loop refinement (Schrödinger, LCC, New York, 2012). This procedure samples alternative backbone conformations and optimizes loop geometry under the OPLS2005 force field,^63^ ensuring a more accurate active-site architecture for subsequent docking.

#### 4.5.3 Ligand Preparation

A series of 26 CYP5122A1 and 37 CYP51 inhibitors (IC_50_ ≤ 10 μM) was compiled from the literature. Then, decoys were generated for each inhibitor via the LUDe server, yielding a total of 1,300 decoys for CYP5122A1 and 1,850 decoys for CYP51. All actives, decoys, and prioritized hits were processed with LigPrep 2.5 to generate low-energy conformers and predict protonation states at pH 7.4 ± 0.5.

#### 4.5.4 Molecular Docking

Grid boxes were established into *x*, *y*, and *z* coordinates of CYP5122A1 (37.64 × −25.58 × −49.06) and CYP51 (37.64 × −25.68 × −49.25) using the receptor grid generation panel of Glide v.5.8. Then, molecular docking calculations were performed on the Maestro workspace Glide v.5.8,^64,65^ employing extra precision (XP) mode. The poses were scored using GlideScore and further optimized using the OPLS2005 force field.^63^ Docking energy terms and scores were exported and used to train a Naive Bayes classifier in KNIME v.5.2.5 with 5-fold cross-validation. The performance of the docking protocol and Bayesian rescoring model was assessed using EF, BEDROC, and AUC metrics.

### 4.6 Experimental Validation

#### 4.6.1 Chemicals

Compounds were purchased from ChemBridge (San Diego, CA, USA), dissolved in 100% dimethyl sulfoxide (DMSO) to achieve a stock concentration of 20 mM, and stored at −20 °C. The chemical structures of all compounds were verified using proton nuclear magnetic resonance (^1^H NMR) spectroscopy or liquid chromatography–mass spectrometry (LC-MS) analysis, which included evaporative light scattering and ultraviolet detectors. This analysis confirmed that all compounds had a minimum purity of 95% (spectra of the compounds are provided in the Figures S2–S19).

#### 4.6.2 Parasite and Cell Culture

*L. infantum* promastigotes were grown at 26 °C in Schneider medium (Sigma), supplemented with 20% heat-inactivated fetal bovine serum (Sigma) and 1% penicillin-streptomycin (Sigma). To ensure maintenance of virulence, the parasites were serially passed through BALB/c mice. THP-1 cells were cultured in RPMI-1640 medium (Sigma-Aldrich), supplemented with 10% heat-inactivated SFB (Sigma) and 1% penicillin-streptomycin (Life Technologies), maintained at a density of 10^6^ cells/mL, and incubated at 37 °C in a 5% CO_2_ atmosphere. For differentiation, THP-1 cells were seeded in 96-well culture plates at a concentration of 1 × 10^4^ cells/well and treated with 100 ng/mL of 4α-phorbol 12-myristate 13-acetate (PMA, Sigma-Aldrich) and incubated for 48 h at 37 °C and 5% CO_2_. Cell viability was previously assessed using the Trypan Blue exclusion method. After stimulation with PMA, the cells exhibited characteristics of macrophages, including morphological changes and adherence to the culture plates. Differentiated THP-1 cells were washed with nonsupplemented RPMI-1640 and given fresh medium without PMA. The cells were rested for 24 h before further treatment.

#### 4.6.3 Promastigote Assay

To evaluate the effect of prioritized compounds on parasite viability, 4 × 10^6^ promastigotes/mL were incubated in the presence of a series of 11 points (0.1% DMSO) for 48 h at 28 °C to a final volume of 200 μL in a 96-well microplate, with each condition tested in technical replicates (≥2 wells per condition). Next, 20 μL of resazurin solution (0.39 mM) was added to each well and incubated for 4 h, and then fluorescence was recorded (570 nm_ex_/595 nm_em_) on a microplate reader SpectraMax M5 (Molecular Devices, Sunnyvale, CA). The IC_50_ values were determined in three independent experiments by fitting concentration–response curves using nonlinear regression with a four-parameter logistic (4PL) model in GraphPad Prism v10.

#### 4.6.4 Intracellular Assay

THP-1 cells differentiated in 96-well culture plates were infected with promastigotes in the stationary phase at a ratio of 30 parasites: 1 cell for 4 h at 37 °C in a final volume of 100 μL. The noninternalized parasites were then removed by washing with RPMI-1640 medium. The compounds were diluted at concentrations of 200–0.7 μM in 100 μL of culture medium per well for serial dilution. Subsequently, 100 μL of the diluted compounds was added to the infected THP-1 cells and incubated for 48 h, with each condition tested in technical replicates (≥2 wells per condition). The number of intracellular parasites per 100 macrophages was counted using light microscopy. The infection rate was determined in relation to the mean of the control wells. The IC_50_ values were determined independently in three separate experiments by fitting concentration–response curves to a 4PL model in GraphPad Prism v10.

#### 4.6.5 Cytotoxicity Assay

THP-1 cells were cultured in 96-well plates at a density of 1 × 10^4^ cells per well in 100 μL of RPMI 1640 medium supplemented with 10% heat-inactivated fetal bovine serum (FBS) and 25 mM HEPES (pH 7.4). Cultures were maintained at 37 °C in a humidified atmosphere containing 5% CO_2_. After 24 h of incubation, the test compounds were prepared in serial dilutions in complete medium, starting at a concentration of 200 μM. Subsequently, 100 μL of each diluted compound solution was added to the corresponding wells containing the cells, with each concentration tested in duplicate wells. Following a 72 h incubation period, resazurin solution (Sigma) was added to each well at a final concentration of 20 μM, and plates were incubated for an additional 4 h under the same conditions. Fluorescence was measured using a SpectraMax M5 microplate reader (Molecular Devices, Sunnyvale, CA, USA) with an excitation wavelength of 570 nm and an emission wavelength of 595 nm. The cytotoxic concentration that reduced cell viability by 50% (CC_50_) was determined based on data from two independent experiments and calculated using nonlinear regression analysis, as previously described for IC_50_ determination.

#### 4.6.6 In Vitro ADME Characterization

ADME profiling was conducted using liquid chromatography coupled with tandem mass spectrometry (LC–MS/MS). The chromatographic separation was carried out on a Prominence UFLC system (Shimadzu Corporation, Kyoto, Japan), connected to an LCMS-8045 triple quadrupole mass spectrometer (Shimadzu Corporation, Kyoto, Japan) with an electrospray ionization.

##### 4.6.6.1 Kinetic Solubility

Kinetic solubility of the test compounds was assessed by preparing 10 mM stock solutions in DMSO, which were then dispensed into two 96-well incubation plates, each in duplicate. For each well, either PBS at pH 7.4 or 2.0 was added to reach a final compound concentration of 250 μM, keeping the DMSO content below 2.5%. The plates were sealed and shaken (200 rpm) at 25 °C for 24 h to allow for equilibrium solubility to be reached. The precipitates on the incubation plate were removed by centrifugation at 3000 rpm for 15 min at 25 °C, and the supernatant fractions were quantified by LC-MS/MS. An intermediate standard solution diluted to 0.5 mM in a 1:1 solution of acetonitrile and water. A calibration curve was prepared for each of the test compounds and controls by diluting several times the intermediate standard solution to reach the desired concentrations of 50, 40, 20, 2, and 1 μM. The resulting equation for the calibration (*y* = *mx* + *b*) was used to calculate the actual concentrations present in the test samples. The chromatogram for analysis was achieved on a Supelco Ascentis Express C18 column (3 cm × 2.1 mm, 5 μm particle size) using water with 0.05% formic acid (A) and acetonitrile with 0.05% formic acid (B) as mobile phase. The mobile phase was eluted in binary gradient mode, and the gradient was as follows: 0 min: 98% A; 1.2 min: 2% A; 2.0 min: 2% A; re-equilibration time: 0.6 min, 98% A. The total run time was 2 min per sample, with an injection volume of 5 μL and a flow rate of 0.6 mL/min.

##### 4.6.6.2 Experimental Determination of Distribution Coefficient (eLogD)

The eLogD was carried out using a chromatographic method based on the analyte retention times within a stationary phase. Separation was performed on a Supelco Ascentis Express RP Amide high-performance liquid chromatography (HPLC) column (5 cm × 2.1 mm, 2.7 μm particle size), utilizing a binary mobile phase system composed of 5% methanol in 10 mM ammonium acetate buffer at pH 7.4 (designated as solvent A), and pure methanol (solvent B). The gradient program was as follows: initial composition at 95% A; shifted to 100% A at 0.3 min; reduced to 0% A by 5.2 min; maintained at 0% A until 5.6 min; returned to 100% A at 5.8 min; and held until the end of the 7 min run. The injection volume for each sample was 5 μL. Test compounds were diluted to a concentration of 1.0 mg/mL in a 1:1 mixture of mobile phases A and B, containing an internal standard at 200 nM. Final DMSO concentration was kept below 2%. To determine compound lipophilicity, each test molecule was injected individually along with a panel of eight reference drugs with known eLogD_7.4_ values ranging from −1.86 to 6.10. These standards included: acyclovir (−1.86), atenolol (0.16), antipyrine (0.38), fluconazole (0.50), prednisone (1.46), ketoconazole (3.83), tolnaftate (5.40), and amiodarone (6.10). A calibration curve was generated by plotting the retention times of these standards against their corresponding eLogD values. The linear regression equation (*y* = *mx* + *b*) obtained from this curve was then used to calculate the eLogD of each test compound.

##### 4.6.6.3 Parallel Artificial Membrane Permeability Assays

To evaluate the effective permeability (Pe) of the compounds, a precoated 96-well BD Gentest PAMPA plate (Corning Gentest #353015) is used. Each well is divided into two chambers, donor and acceptor, separated by a triple-layer phospholipid membrane constructed on a porous filter. Solutions of the compounds are prepared by diluting their respective stock solutions (10 mM in DMSO) in phosphate-buffered saline (PBS) at pH 6.5, yielding a final concentration of 10 μM (<1% DMSO, v/v). The solutions are then added to the donor portion of the plate (300 μL/well) in triplicate, while the acceptor portion received only PBS pH 7.4 (200 μL/well). The donor and acceptor portions are subsequently assembled, and the system is incubated for 5 h at 37 °C and 100 rpm. Samples of the initial donor solution (T_zero_) are collected and transferred to an analysis plate (10 μL), followed by the addition of stop solution (300 μL) [10% ultrapure water (type 1) and 90% methanol (HPLC grade ≥ 99.9%):acetonitrile (HPLC grade ≥ 99.9%) (50:50) + 50 nM internal standard] and PBS buffer pH 6.5 (60 μL). After the incubation period, samples are collected from the donor (10 μL) and acceptor (80 μL) wells and added to the analysis plate containing stop solution (300 μL) and PBS buffer pH 6.5 (60 μL). Final compound concentrations in the donor, acceptor, and T_zero_ wells are quantified by LC-MS/MS. The chromatogram for analysis was achieved on Supelco Ascentis express C18 column (3 cm × 2.1 mm, 5 μM). The mobile phases consisted of water +0.1% formic acid (A) and acetonitrile +0.1% formic acid (B). Mobile phase was eluted in binary gradient mode, and the gradient was as follows: 0 min, 95% A; 0.05 min. 95% A; 0.3 min: 2% A; 0.7 min: 2% A; 0.8 min: 95% A; 1.15 min 95% A; 2.0 min 95% A. The run time was 2 min, and the sample injection volume was 10 μL and flow rate of 0.7 mL/min. The results were used to calculate an effective permeability (Pe) value. All PAMPA assay was performed in triplicates.

##### 4.6.6.4 Human and Mouse Liver Microsomal Stability Assay

Metabolic stability of the test compounds was assessed using pooled human liver microsomes (20 mg/mL, GIBCO) and CD1 mouse liver microsomes (20 mg/mL, GIBCO). Compounds were diluted to a final concentration of 0.5 μM and incubated with microsomal protein at 0.25 mg/mL in phosphate-buffered saline (PBS) at pH 7.4. The DMSO content in the incubation mixture was maintained below 1%. The metabolic reaction was initiated by introducing NADPH as a cofactor at a concentration of 0.5 μM. Aliquots were taken at defined time intervals: 0 (immediately after NADPH addition), 5, 10, 20, 30, and 60 min. Reactions were halted by the addition of a quenching solvent consisting of a 1:1 mixture of acetonitrile and methanol containing an internal standard at 50 nM. Following quenching, samples were centrifuged at 3500 rpm for 30 min to pellet the precipitated microsomal proteins. The resulting supernatants were analyzed via LC–MS/MS. Quantification was performed based on the peak area ratio (PAR) of the analyte to internal standard, with the signal at time zero defined as 100%. The percentage of parent compound remaining at each time point was calculated accordingly. Using the plot of % remaining versus incubation time, the degradation rate constant (*k*) was determined via nonlinear regression. From this, the half-life (*T*_1/2_ = ln(2)/*k*, in minutes) and intrinsic clearance (CL_int_ = *k* × 1000/0.25, in μL/min/mg protein) were calculated. Chromatographic analysis was carried out using a Supelco Ascentis Express C18 column (3 cm × 2.1 mm, 5 μm particle size). The mobile phases were composed of water with 0.1% formic acid (A) and acetonitrile with 0.1% formic acid (B). A binary gradient was applied as follows: 0–0.05 min, 95% A; 0.3:0.7 min, 2% A; 0.8:2.0 min, re-equilibration at 95% A. The total run time was 2 min per sample, with an injection volume of 10 μL and a flow rate of 0.7 mL/min. All metabolic stability assays were conducted in triplicate.

## Supplementary Material













## Data Availability

All data sets and source code used in this study are publicly available through GitHub. The repository for GNN development and benchmarking is accessible at https://github.com/LCi-UFG/HolisticGNN. The repository containing the scripts and data for target fishing validation is available at https://github.com/LCi-UFG/BayesDocking.

## Notes

Baselines for random and scaffold split for imbalanced and balanced data sets; attentional explainability of predicted antileishmanial activity; nearest neighbors of each putative hit; ^1^H NMR and/or LC-MS spectra for test compounds; and *in vitro* ADME assays for best hits (PDF)
Kinetic solubility assay (XLSX)
Determination of experimental LogD (eLogD) (XLSX)
Parallel artificial membrane permeability assay (PAMPA) (XLSX)
Metabolic stability in mouse liver microsomes (XLSX)
Metabolic stability in human liver microsomes (XLSX)
