# Supplementary material for: Multiscale-Aware Graph Embedding Approach Uncovers LC-61, a Potent Anti-Leishmania infantum Compound
Source: J Chem Inf Model. 2026 Mar 17;66(7):3643–59. doi: 10.1021/acs.jcim.5c02947 (PMC13080969; doi:10.1021/acs.jcim.5c02947)
Supplement: Supplementary file 1 [file ci5c02947_si_001.pdf]

Supporting Information

Multiscale-Aware Graph Embedding Approach Uncovers LC-61, a Potent Anti–*Leishmania infantum* Compound

Vinícius Alexandre Fiaia Costa,[‡,1] Alexandra Maria dos Santos Carvalho,[‡,2] Rafael Consolin Chelucci,[3] Felipe da Silva Mendonça de Melo,[2] Gustavo Santos Sandes Felizardo,[1] Clarissa Alves Carneiro Bernardes,[1] Holli-Joi Martin,[4] Rodolpho de Campos Braga,[6] Sébastien Charneau,[5] Eugene N. Muratov,[4] Adriano Defini Andricopulo,[3] Izabela Marques Dourado Bastos,[2] Bruno Junior Neves[1,*]

[1] Laboratory of Cheminformatics, Faculty of Pharmacy, Federal University of Goiás, Goiânia, Brazil; [2] Pathogen-Host Interface Laboratory, Department of Cell Biology, Institute of Biological Sciences, University of Brasilia, Brasilia, Brazil; [3] Laboratory of Medicinal and Computational Chemistry, Sao Carlos Institute of Physics, University of Sao Paulo, IFSC – USP, 13566-590, Sao Carlos, SP, Brazil; [4] Laboratory for Molecular Modeling, UNC Eshelman School of Pharmacy, University of North Carolina at Chapel Hill, North Carolina, USA; [5] Laboratory of Protein Chemistry and Biochemistry, Department of Cell Biology, Institute of Biological Sciences, University of Brasilia, Brasilia, Brazil; [6] InsilicAll Ltda, São Paulo, Brazil

*Author for correspondence: brunoneves@ufg.br

‡ Authors contributed equally

CONTENTS

1. Benchmark analysis 2
2. Attention scores 5
3. Structural novelty analysis 6
4. Counterfactual rules 8
5. ^{1}H NMR and LC-MS spectra for test compounds 10

1. Benchmark analysis

Table S1. Overall predictive performance of evaluated models on the imbalanced dataset.

Method	Set	Threshold	Accuracy	Recall	Specificity	G-mean	MCC	PR-AUC	AUC
Random Split									
**RF**	Training	0.50	0.90	0.35	0.99	0.59	0.51	0.73	0.92
	Validation	0.50	0.87	0.16	1.00	0.40	0.34	0.67	0.89
**SVM**	Training	0.50	0.86	0.04	1.00	0.20	0.17	0.64	0.86
	Validation	0.50	0.88	0.10	1.00	0.32	0.30	0.53	0.83
**D-MPNN**	Training	0.50	0.85	0.01	1.00	0.08	0.07	0.43	0.81
	Validation	0.50	0.83	0.00	1.00	0.00	0.00	0.45	0.83
	Test	0.50	0.86	0.00	1.00	0.00	0.00	0.41	0.83
**MPNN**	Training	0.50	0.87	0.18	0.99	0.43	0.34	0.61	0.90
	Validation	0.50	0.85	0.17	0.99	0.41	0.32	0.65	0.89
	Test	0.50	0.88	0.20	0.98	0.44	0.32	0.50	0.81
**GIN**	Training	0.50	0.89	0.41	0.97	0.63	0.49	0.62	0.89
	Validation	0.50	0.87	0.33	0.97	0.57	0.43	0.55	0.84
	Test	0.50	0.90	0.40	0.98	0.63	0.52	0.56	0.91
**GAT**	Training	0.50	0.87	0.28	0.97	0.52	0.35	0.53	0.87
	Validation	0.50	0.84	0.25	0.96	0.49	0.29	0.44	0.83
	Test	0.50	0.86	0.20	0.97	0.44	0.25	0.46	0.89
Scaffold Split									
**RF**	Training	0.50	0.91	0.47	0.98	0.68	0.57	0.71	0.93
	Test	0.50	0.84	0.49	0.91	0.67	0.41	0.48	0.85
**SVM**	Training	0.50	0.86	0.05	1.00	0.22	0.19	0.59	0.86
	Test	0.50	0.85	0.00	1.00	0.00	0.00	0.22	0.56
**D-MPNN**	Training	0.50	0.86	0.05	1.00	0.23	0.19	0.48	0.83
	Validation	0.50	0.80	0.00	0.98	0.00	-0.06	0.37	0.79
	Test	0.50	0.86	0.14	0.98	0.37	0.24	0.34	0.69
**MPNN**	Training	0.50	0.88	0.22	0.99	0.47	0.38	0.61	0.88
	Validation	0.50	0.82	0.08	0.98	0.28	0.14	0.45	0.82
	Test	0.50	0.85	0.00	1.00	0.00	0.00	0.54	0.82
**GIN**	Training	0.50	0.87	0.09	1.00	0.30	0.26	0.51	0.85
	Validation	0.50	0.85	0.15	1.00	0.39	0.36	0.74	0.93
	Test	0.50	0.86	0.05	1.00	0.22	0.20	0.23	0.50
**GAT**	Training	0.50	0.86	0.00	1.00	0.00	0.00	0.35	0.77
	Validation	0.50	0.82	0.00	1.00	0.00	0.00	0.49	0.83
	Test	0.50	0.85	0.00	1.00	0.00	0.00	0.48	0.76

Table S2. Overall predictive performance of evaluated models on the imbalanced dataset after threshold-moving calibration.

Method	Set	Threshold	Accuracy	Recall	Specificity	G-mean	MCC	PR-AUC	AUC
Random Split									
**RF**	Training	0.19	0.86	0.83	0.86	0.85	0.57	0.73	0.92
	Test	0.19	0.85	0.75	0.86	0.81	0.53	0.67	0.89
**SVM**	Training	0.12	0.79	0.80	0.78	0.79	0.45	0.64	0.86
	Test	0.12	0.76	0.85	0.74	0.79	0.43	0.53	0.83
**D-MPNN**	Training	0.28	0.83	0.56	0.88	0.70	0.40	0.43	0.81
	Validation	0.28	0.87	0.75	0.89	0.82	0.58	0.45	0.83
	Test	0.28	0.83	0.45	0.89	0.63	0.32	0.41	0.83
**MPNN**	Training	0.09	0.81	0.89	0.79	0.84	0.52	0.61	0.90
	Validation	0.17	0.86	0.88	0.86	0.87	0.62	0.65	0.89
	Test	0.17	0.82	0.75	0.83	0.79	0.46	0.50	0.81
**GIN**	Training	0.13	0.80	0.85	0.79	0.82	0.50	0.62	0.89
	Validation	0.11	0.77	0.83	0.76	0.79	0.46	0.55	0.84
	Test	0.11	0.77	0.90	0.75	0.82	0.47	0.56	0.91
**GAT**	Training	0.12	0.79	0.83	0.79	0.81	0.47	0.53	0.87
	Validation	0.13	0.80	0.83	0.80	0.82	0.51	0.44	0.83
	Test	0.13	0.84	0.90	0.83	0.86	0.57	0.46	0.89
Scaffold Split									
**RF**	Training	0.15	0.82	0.86	0.82	0.84	0.53	0.71	0.93
	Test	0.15	0.71	0.85	0.69	0.77	0.41	0.48	0.85
**SVM**	Training	0.11	0.78	0.80	0.78	0.79	0.44	0.59	0.86
	Test	0.11	0.55	0.43	0.57	0.49	0.00	0.22	0.56
**D-MPNN**	Training	0.31	0.84	0.46	0.90	0.64	0.36	0.48	0.83
	Validation	0.31	0.71	0.85	0.68	0.76	0.42	0.37	0.79
	Test	0.31	0.81	0.24	0.90	0.46	0.15	0.34	0.69
**MPNN**	Training	0.14	0.80	0.81	0.79	0.80	0.46	0.61	0.88
	Validation	0.23	0.77	1.00	0.72	0.85	0.56	0.45	0.82
	Test	0.23	0.78	0.57	0.81	0.68	0.32	0.54	0.82
**GIN**	Training	0.10	0.78	0.76	0.78	0.77	0.42	0.51	0.85
	Validation	0.17	0.84	0.92	0.82	0.87	0.62	0.74	0.93
	Test	0.17	0.78	0.14	0.89	0.36	0.03	0.23	0.50
**GAT**	Training	0.08	0.72	0.73	0.72	0.72	0.33	0.35	0.77
	Validation	0.13	0.72	1.00	0.66	0.81	0.51	0.49	0.83
	Test	0.13	0.84	0.38	0.92	0.59	0.32	0.48	0.76

Table S3. Overall predictive performance of evaluated models on the balanced dataset.

Method	Set	Threshold	Accuracy	Recall	Specificity	G-mean	MCC	PR-AUC	AUC
Random Split									
**RF**	Training	0.50	0.81	0.74	0.88	0.81	0.63	0.91	0.92
	Test	0.50	0.81	0.75	0.87	0.80	0.62	0.87	0.88
**SVM**	Training	0.50	0.77	0.71	0.81	0.76	0.52	0.80	0.84
	Test	0.50	0.79	0.71	0.86	0.78	0.58	0.89	0.88
**D-MPNN**	Training	0.50	0.76	0.74	0.78	0.76	0.52	0.78	0.83
	Validation	0.50	0.77	0.76	0.77	0.77	0.53	0.61	0.77
	Test	0.50	0.80	0.76	0.84	0.80	0.60	0.80	0.85
**MPNN**	Training	0.50	0.80	0.77	0.83	0.80	0.61	0.86	0.89
	Validation	0.50	0.79	0.79	0.80	0.80	0.58	0.76	0.86
	Test	0.50	0.82	0.78	0.85	0.81	0.63	0.92	0.92
**GIN**	Training	0.50	0.84	0.75	0.91	0.82	0.67	0.91	0.93
	Validation	0.50	0.82	0.71	0.89	0.79	0.62	0.80	0.87
	Test	0.50	0.82	0.76	0.88	0.82	0.65	0.91	0.91
**GAT**	Training	0.50	0.82	0.73	0.90	0.81	0.64	0.87	0.90
	Validation	0.50	0.83	0.78	0.87	0.82	0.65	0.78	0.86
	Test	0.50	0.85	0.79	0.90	0.85	0.70	0.88	0.89
Scaffold Split									
**RF**	Training	0.50	0.84	0.75	0.91	0.83	0.67	0.92	0.93
	Test	0.50	0.75	0.65	0.82	0.73	0.48	0.66	0.76
**SVM**	Training	0.50	0.77	0.72	0.81	0.76	0.53	0.82	0.85
	Test	0.50	0.68	0.38	0.87	0.58	0.30	0.65	0.72
**D-MPNN**	Training	0.50	0.79	0.77	0.81	0.79	0.58	0.82	0.85
	Validation	0.50	0.63	0.65	0.62	0.63	0.26	0.55	0.67
	Test	0.50	0.73	0.40	0.93	0.61	0.41	0.67	0.72
**MPNN**	Training	0.50	0.76	0.63	0.88	0.74	0.53	0.78	0.82
	Validation	0.50	0.70	0.66	0.73	0.70	0.40	0.66	0.71
	Test	0.50	0.73	0.44	0.91	0.63	0.40	0.64	0.66
**GIN**	Training	0.50	0.63	0.47	0.76	0.60	0.24	0.66	0.71
	Validation	0.50	0.60	0.29	0.84	0.49	0.15	0.55	0.49
	Test	0.50	0.67	0.38	0.85	0.57	0.27	0.58	0.67
**GAT**	Training	0.50	0.71	0.56	0.83	0.68	0.41	0.72	0.75
	Validation	0.50	0.57	0.31	0.77	0.49	0.09	0.57	0.59
	Test	0.50	0.68	0.36	0.87	0.56	0.28	0.57	0.66

2. Attention scores

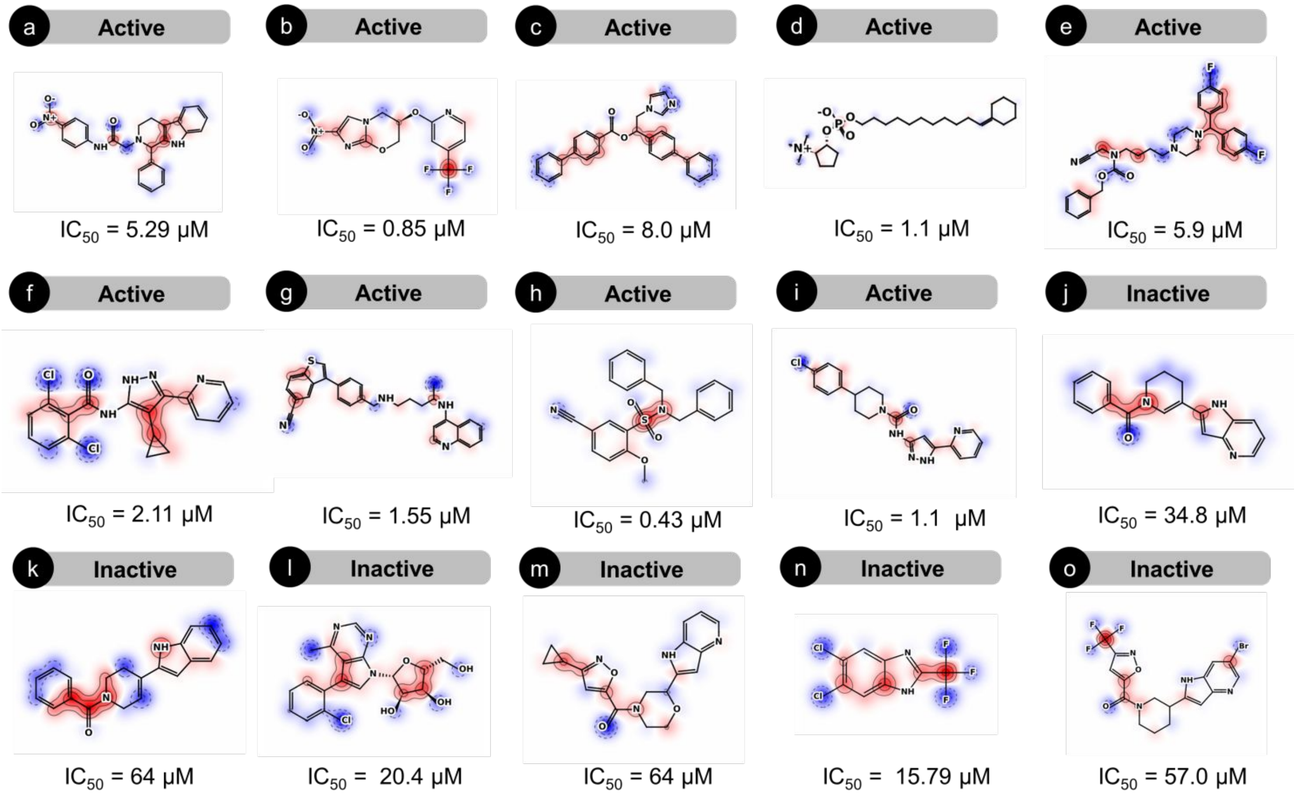

Figure S1. Attentional explainability of predicted antileishmanial activity. (**a-i**) shows attention-weight visualizations for selected actives while (**j-o**) shows attention-weight visualizations for selected inactives. Red contours indicate regions contributing positively to antileishmanial activity, whereas blue contours indicate regions contributing negatively.

3. Structural novelty analysis

Table S4. Nearest neighbors identified in the ChEMBL database for the putative hits reported in this study.

Compound	Structure	Nearest Neighbor	Percent Inhibition (%) / IC_{50} (μM)	Tanimoto Score	ChEMBL Document ID
LC-58	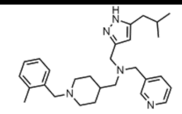	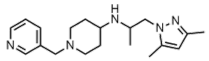	19.59%	0.90	CHEMBL3988442
LC-59	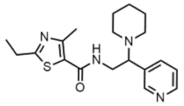	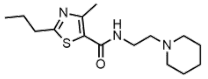	20.27%	0.92	CHEMBL3988442
LC-60	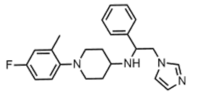	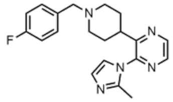	4.173%	0.89	CHEMBL3988442
LC-61	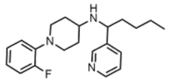	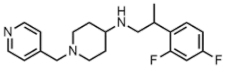	10.91%	0.87	CHEMBL3988442
LC-62	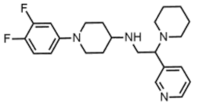	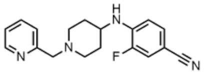	-12.41%	0.91	CHEMBL3988442
LC-63	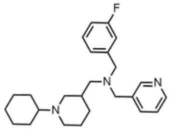	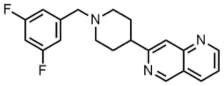	29.54%	0.92	CHEMBL3988442
LC-64	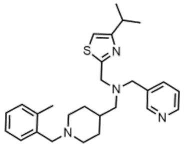	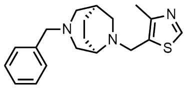	-7.937%	0.89	CHEMBL3988442

LC-65	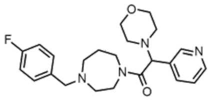	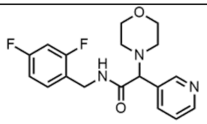	39.19%	0.88	CHEMBL3988442
LC-66	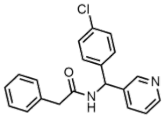	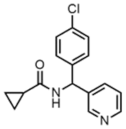	32.37%	0.75	CHEMBL3988442
LC-67	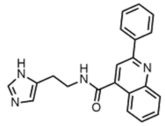	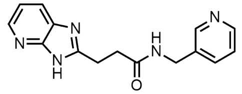	3.71%	0.86	CHEMBL3988442
LC-68	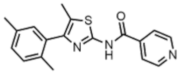	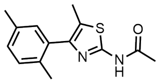	31.6 μM	0.94	CHEMBL3431042
LC-69	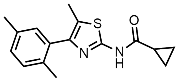	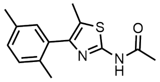	31.6 μM	0.89	CHEMBL3431042
LC-70	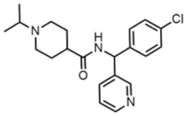	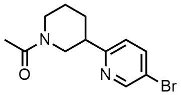	5.521%	0.81	CHEMBL3988442
LC-71	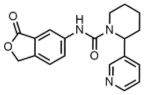	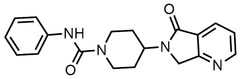	15%	0.87	CHEMBL3988442
LC-72	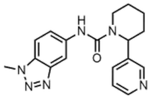	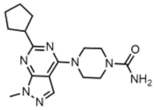	-21.76%	0.87	CHEMBL3988442
LC-73	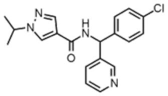	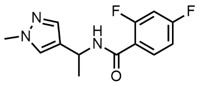	11.9%	0.78	CHEMBL3988442
LC-74	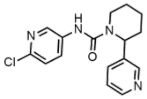	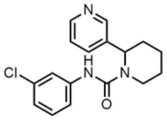	3.16 μM	0.97	CHEMBL3430912

LC-75	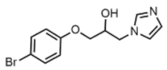	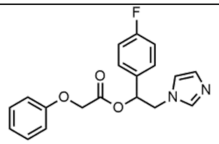	17.3 µM	0.79	CHEMBL2448688

4. Counterfactual rules

Table S5. Rulebook of chemically valid perturbations used for counterfactual explainability.

Rule family	Chemical scope	Typical operation	Examples of representative fragments/motifs	Interpretation in counterfactual maps
**SP2_POLAR_ALL**	Polar substituents attached to sp^2 environments	Isosteric replacement among polar substituents	F, OH, SH, NH_2, NHMe, NMe_2, CF_2, OCF_2, NO_2	Probes how π-conjugated polar decoration modulates prediction
**SP3_POLAR_ALL**	Polar substituents attached to sp^3 environments	Isosteric replacement among polar substituents	F, OH, SH, NH_2, NHMe, NMe_2, CF_2, OCF_2, NO_2	Quantifies local polar effects in saturated regions
**SP2_APOLAR_ALL**	Apolar substituents attached to sp^2 environments	Isosteric replacement among apolar substituents	Me, Cl, Br, I, OMe, Et, iPr, tBu, CF_3, OCF_3, CCl_3, SMe	Probes how π-conjugated hydrophobic/steric decoration modulates prediction
**SP3_APOLAR_ALL**	Apolar substituents attached to sp^3 environments	Isosteric replacement among apolar substituents	Me, Cl, Br, I, OMe, Et, iPr, tBu, CF_3, OCF_3, CCl_3, SMe	Quantifies local hydrophobic/steric effects in saturated regions
**SP2_REACTIVE_ALL**	Electrophilic/reactive fragments in sp^3 settings	Reactive-group insertion/replacement compatible with sp^2 topology	CN, CHO, NO_2, NCO, N_3, N_2, vinyl-F, vinyl-Cl, vinyl-Br, vinyl-I	Assesses effect of introducing latent reactivity in π-conjugated motifs
**SP3_REACTIVE_ALL**	Electrophilic/reactive fragments in sp^3 settings	Reactive-group swap around tetrahedral anchors	CN, CHO, NO_2, NCO, N_3, N_2, vinyl-F, vinyl-Cl, vinyl-Br, vinyl-I	Assesses effect of introducing latent reactivity in saturated/aliphatic motifs
**REDOX_FAMILY**	Oxidation-state–related local chemistry	Redox-like toggles among chemically related states	Alcohol↔carbonyl, thiol ↔ thiocarbonyl, selenol ↔ selenocarbonyl	Isolates contribution of oxidation-state shifts

**ACYL_FAMILY_ALL**	Carbonyl-derived acyl chemistry	Interconversion among acyl-like motifs	Carboxylic acid, ester, ketone, thioketone, selenoketone, thioester	Measures carbonyl-class dependence of prediction
**AMIDE_FAMILY_ALL**	Amide/amide-like nitrogen-containing carbonyl derivatives	Interconversion among amide-like motifs	Amide, thioamide, selenoamide, urea, thiourea, selenourea, guanidine, amidine	Measures contribution of H-bond donor/acceptor amide variants
**CARBAMATE_FAMILY_ALL**	Carbonate/carbamate-related motifs	Replacement within carbonate/carbamate-like motifs	Ester, carbonate, carbamate, thiocarbamate, selenocarbamate, thioester	Evaluates role of masked acyl/N–O/S/Se functionality patterns
**SULFURE_FAMILY_ALL**	Sulfur-centered functional families (including high-valent S)	Sulfur-functional interconversion	Sulfoxide, sulfone, sulfonamide, sulfonate, sulfilimine, sulfoximine, sulfonimidamide	Quantifies sulfur oxidation/neighbor chemistry impact
**PHOSPHORUS_FAMILY_ALL**	Phosphorus-centered functional chemistry	Phosphoryl/phosphonate-like motif replacement	Phosphorothioate, phosphorosulfate, phosphinate, phosphonate, phosphate, phosphorofluoridate, phosphorosulfonamide, phosphorocyanidate, phosphorochloridate	Captures contribution of phosphorus chemistry (often charge-sensitive)
**POLYVALENT_FAMILY_ALL**	polyvalent motifs composed by N^+, P, As, Si, and B	Interconversion among permitted valence states of polyvalent atoms	Phosphonium-like, quaternary ammonium-like, arsonium-like, silyl/silicon-centered, borane-like	Captures contribution of central polyvalent atom (including effective valence expansion/contraction)
**TOGGLE_CHARGE_FAMILY_ALL**	Formal charge states	Local charge toggling under valence-admissible states	Neutral ↔ cationic ↔ anionic states of amines, carboxylic acid, guanidine	Directly probes charge contribution
**DIARYL_FAMILY_ALL**	Biaryl/diaryl-like substructures	Inter-aryl linkage and aryl substitution pattern perturbations	Hydrazine, hydrazone, acyl-hydrazine, oxime, hydroxamic acid, peroxide, disulfide, diarsine	Probes sensitivity to heteroatom-linker identity and lability in connecting motifs
**TOGGLE_RING_FAMILY_ALL**	Ring membership state	Aromaticity-state toggling on ring atoms	Aromatic ↔ Aliphatic rings (e.g., 5- and 6-member contexts)	Tests chemical dependence on aromaticity/conjugation state in intact rings
**RING_FAMILY_ALL**	Equivalent ring membership	Isosteric replacements in ring-level	Phenyl, pyridyl, pyrimidinyl, pyrazinyl, thienyl, piperidinyl, piperazinyl, morpholinyl	Maps dependence on ring identity and aromatic character
**BOND_FAMILY_ALL**	Bond order/linkage type	Inter-aryl linkage and aryl substitution pattern perturbations	Single ↔ double ↔ triple or equivalent admissible transitions	Isolates contribution from local saturation, unsaturation, connectivity

5. ^{1}H NMR and LC-MS spectra for test compounds

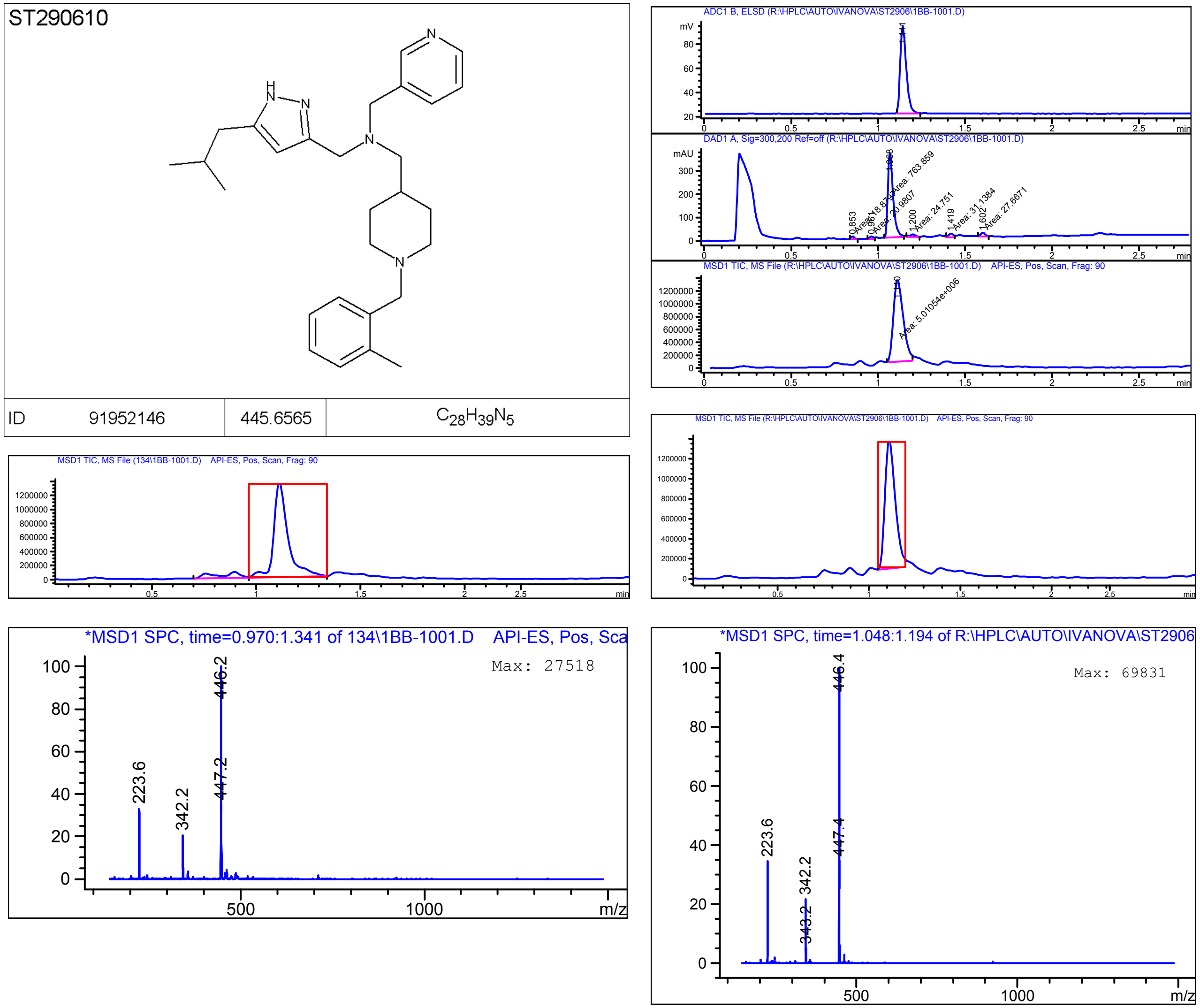

Figure S2. Chromatographic and mass spectra profiles obtained via LC-MS for compound ({1-[(2-methylphenyl)methyl]piperidin-4-yl}methyl)({[5-(2-methylpropyl)-1H-pyrazol-3-yl]methyl})[(pyridin-3-yl)methyl]amine (LC-58).

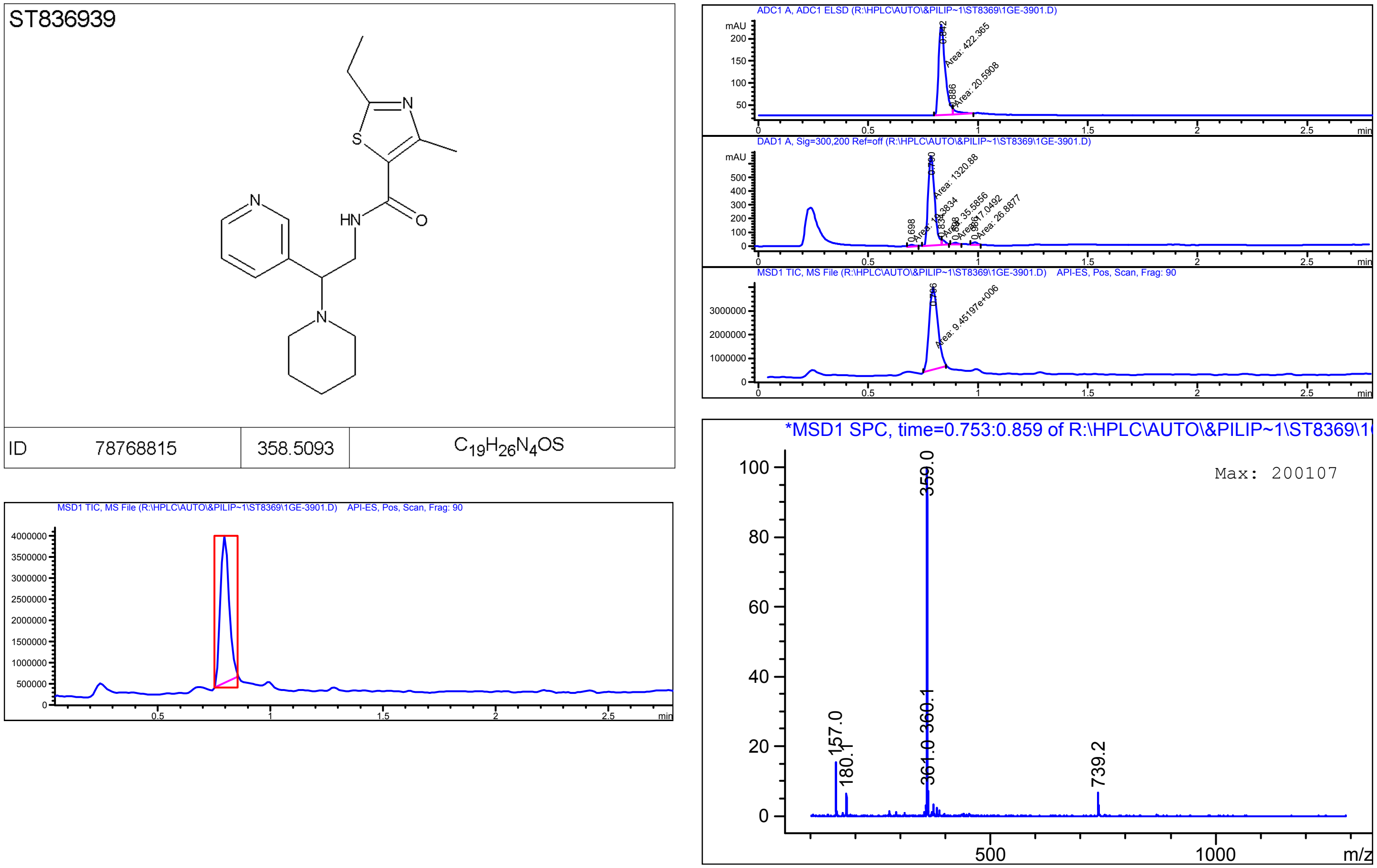

Figure S3. Chromatographic and mass spectra profiles obtained via LC-MS for compound 2-ethyl-4-methyl-N-[2-(piperidin-1-yl)-2-(pyridin-3-yl)ethyl]-1,3-thiazole-5-carboxamide (LC-59).

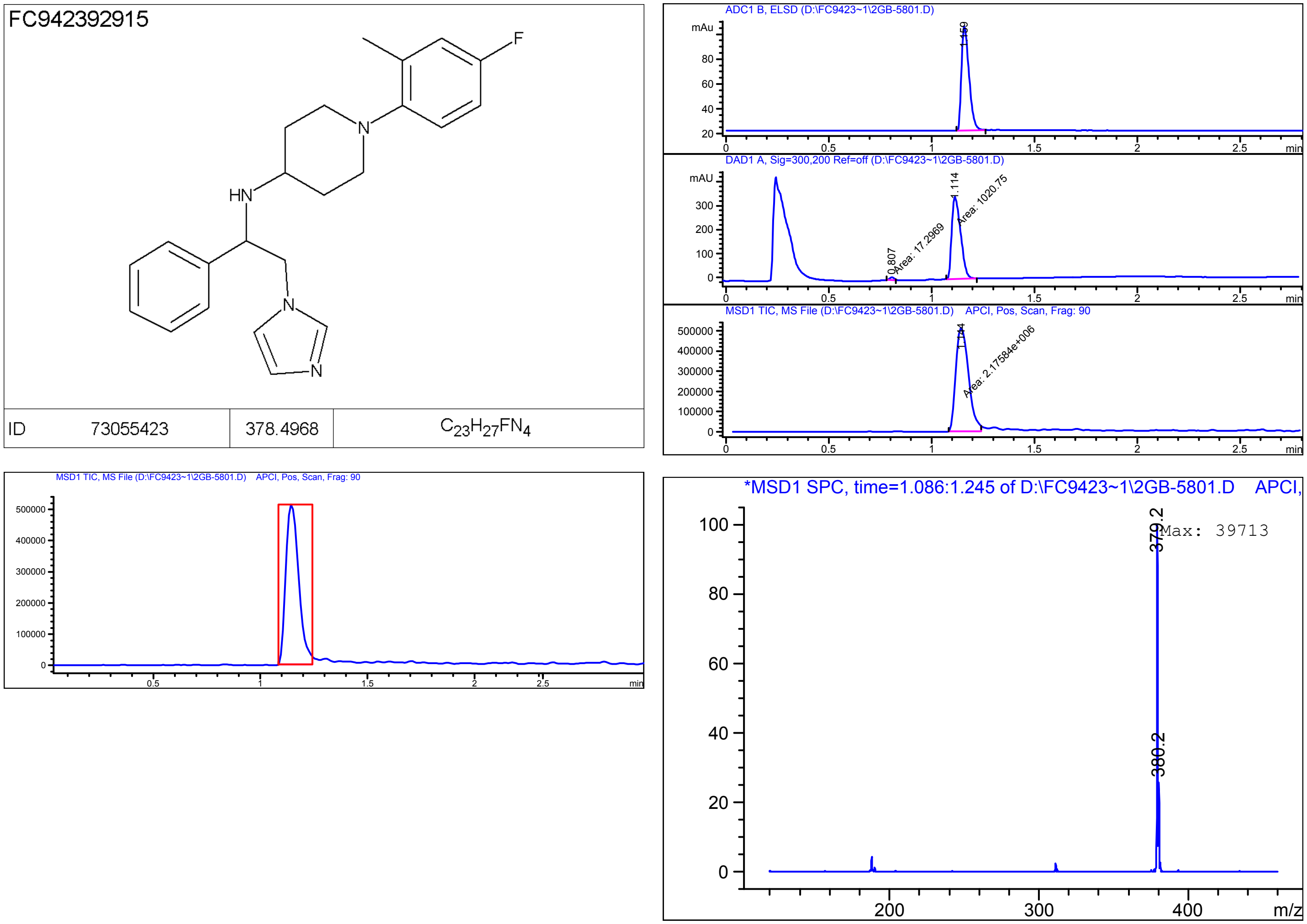

Figure S4. Chromatographic and mass spectra profiles obtained via LC-MS for 1-(4-fluoro-2-methylphenyl)-N-[2-(1H-imidazol-1-yl)-1-phenylethyl]piperidin-4-amine (LC-60).

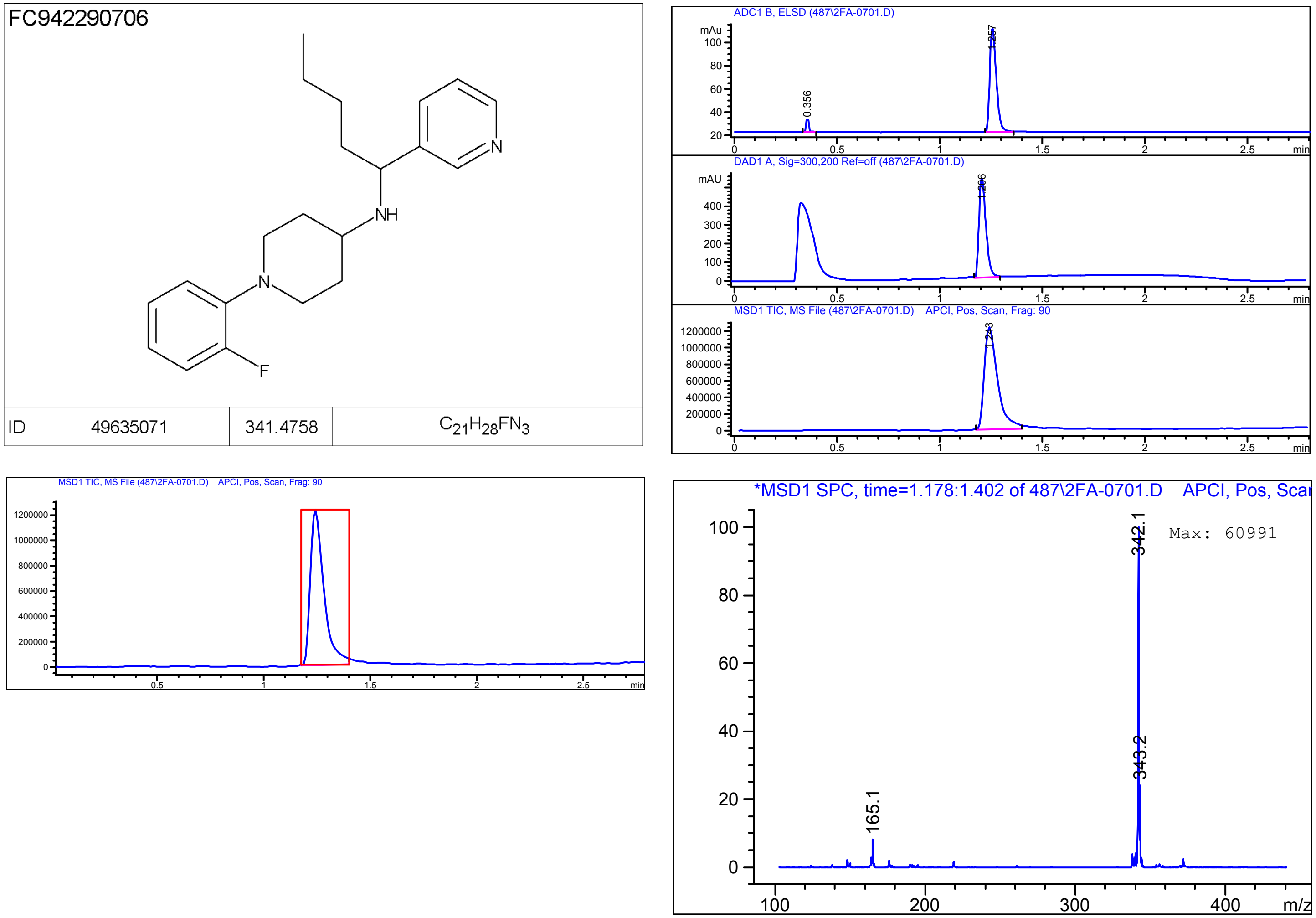

Figure S5. Chromatographic and mass spectra profiles obtained via LC-MS for 1-(2-fluorophenyl)-N-[1-(pyridin-3-yl)pentyl]piperidin-4-amine (LC-61).

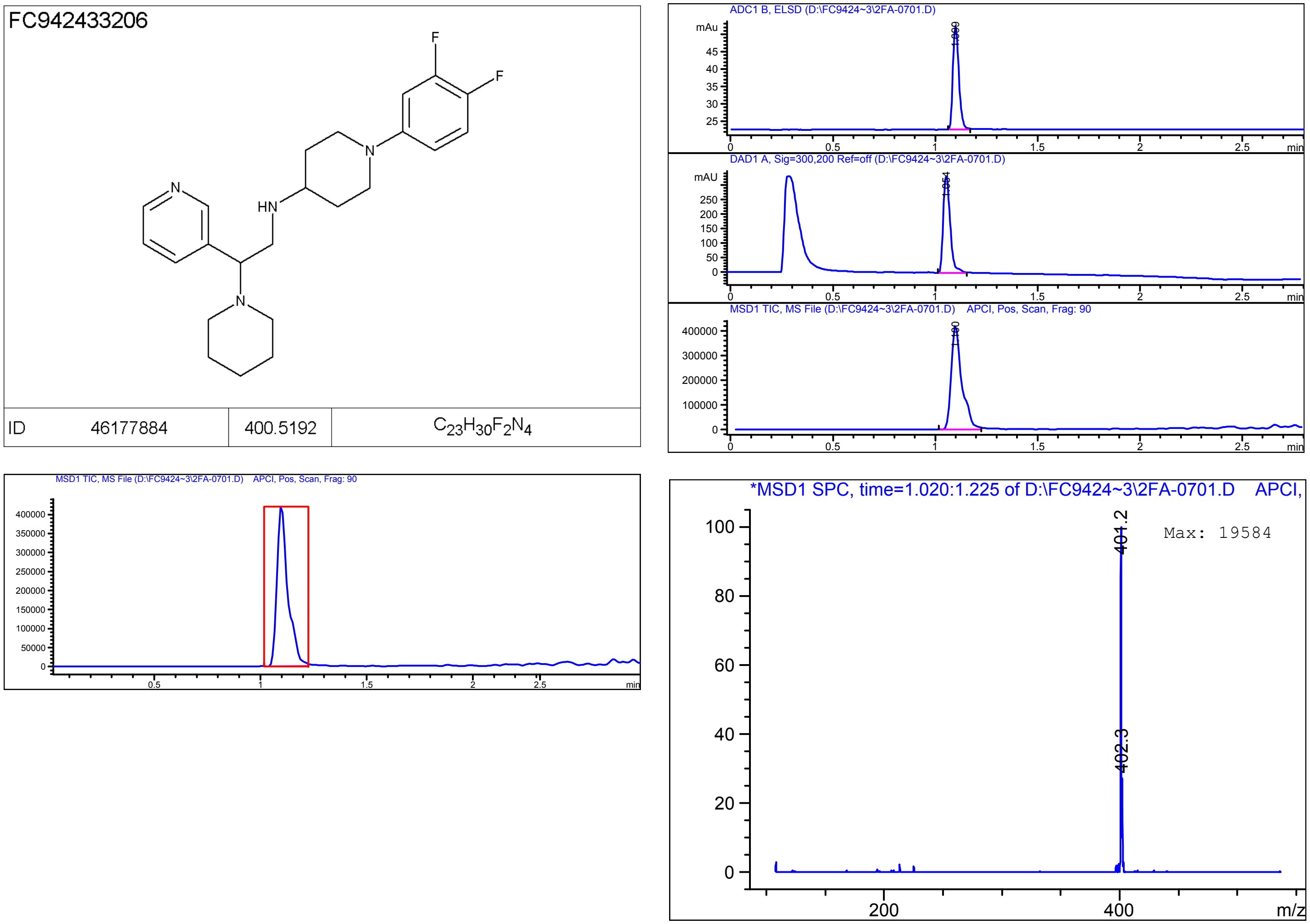

Figure S6. Chromatographic and mass spectra profiles obtained via LC-MS for 1-(3,4-difluorophenyl)-N-[2-(piperidin-1-yl)-2-(pyridin-3-yl)ethyl]piperidin-4-amine (LC-62).

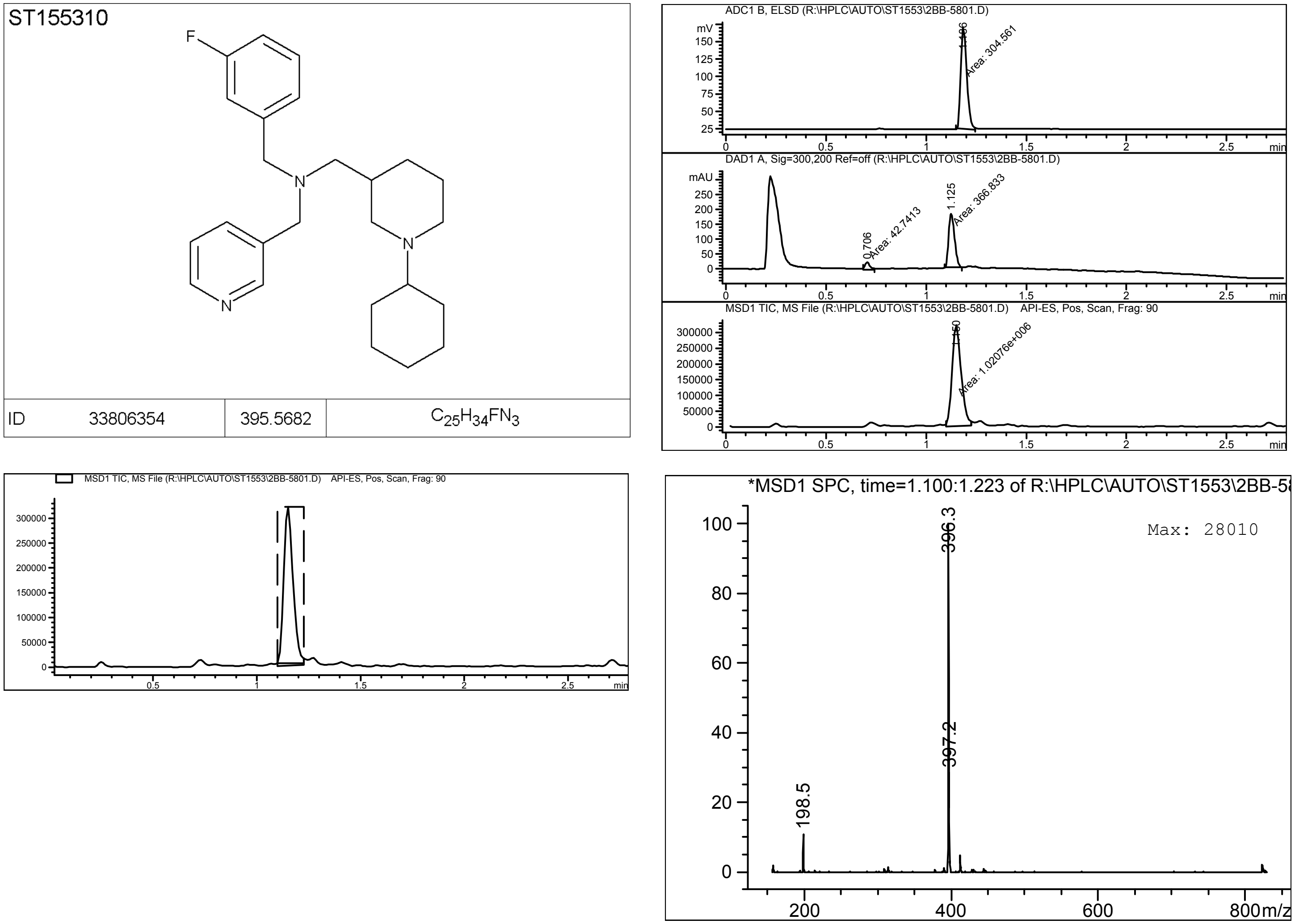

Figure S7. Chromatographic and mass spectra profiles obtained via LC-MS for [(1-cyclohexylpiperidin-3-yl)methyl][(3-fluorophenyl)methyl][(pyridin-3-yl)methyl]amine (LC-63).

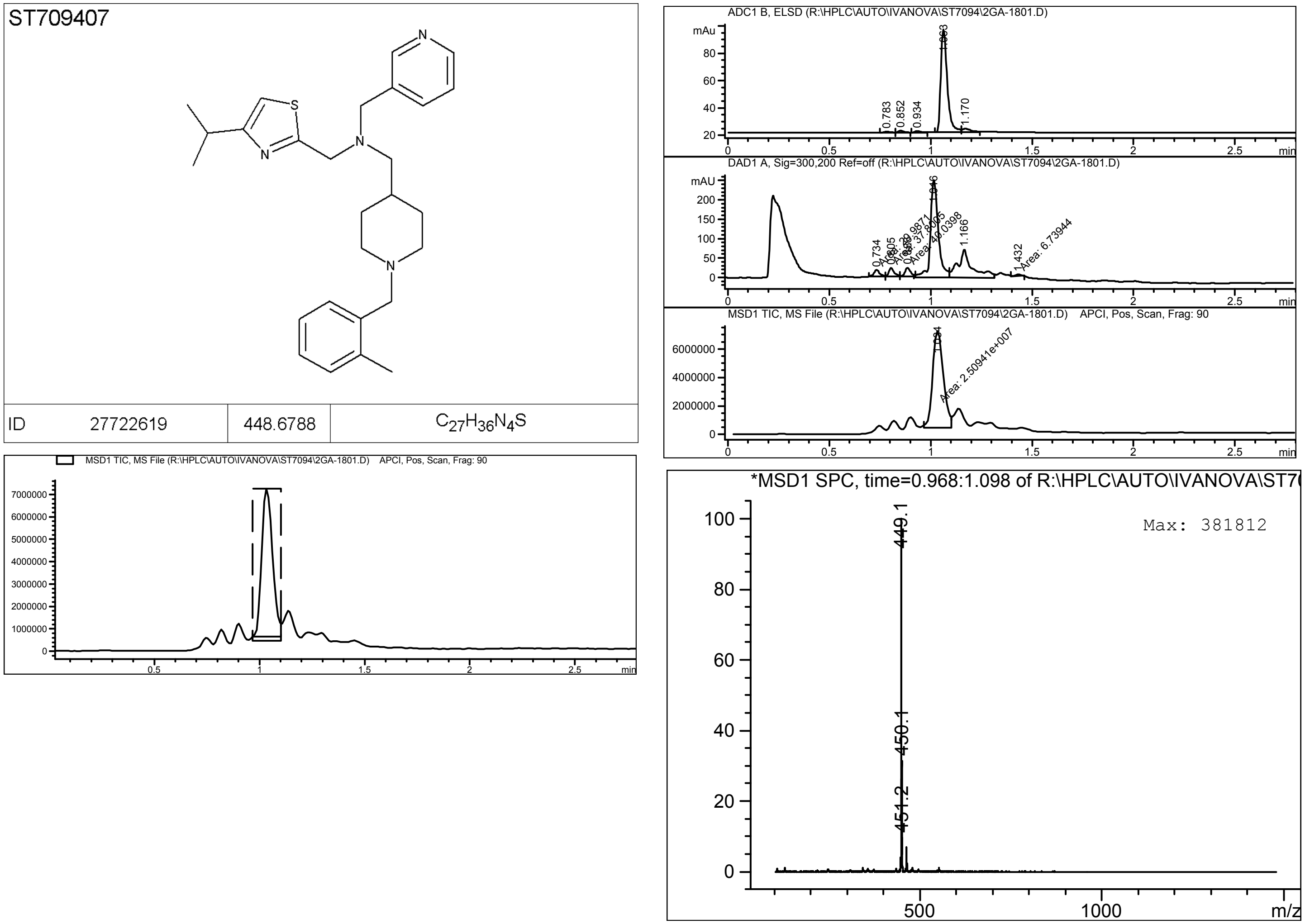

Figure S8. Chromatographic and mass spectra profiles obtained via LC-MS for ({1-[(2-methylphenyl)methyl]piperidin-4-yl}methyl)({[4-(propan-2-yl)-1,3-thiazol-2-yl]methyl})[(pyridin-3-yl)methyl]amine (LC-64).

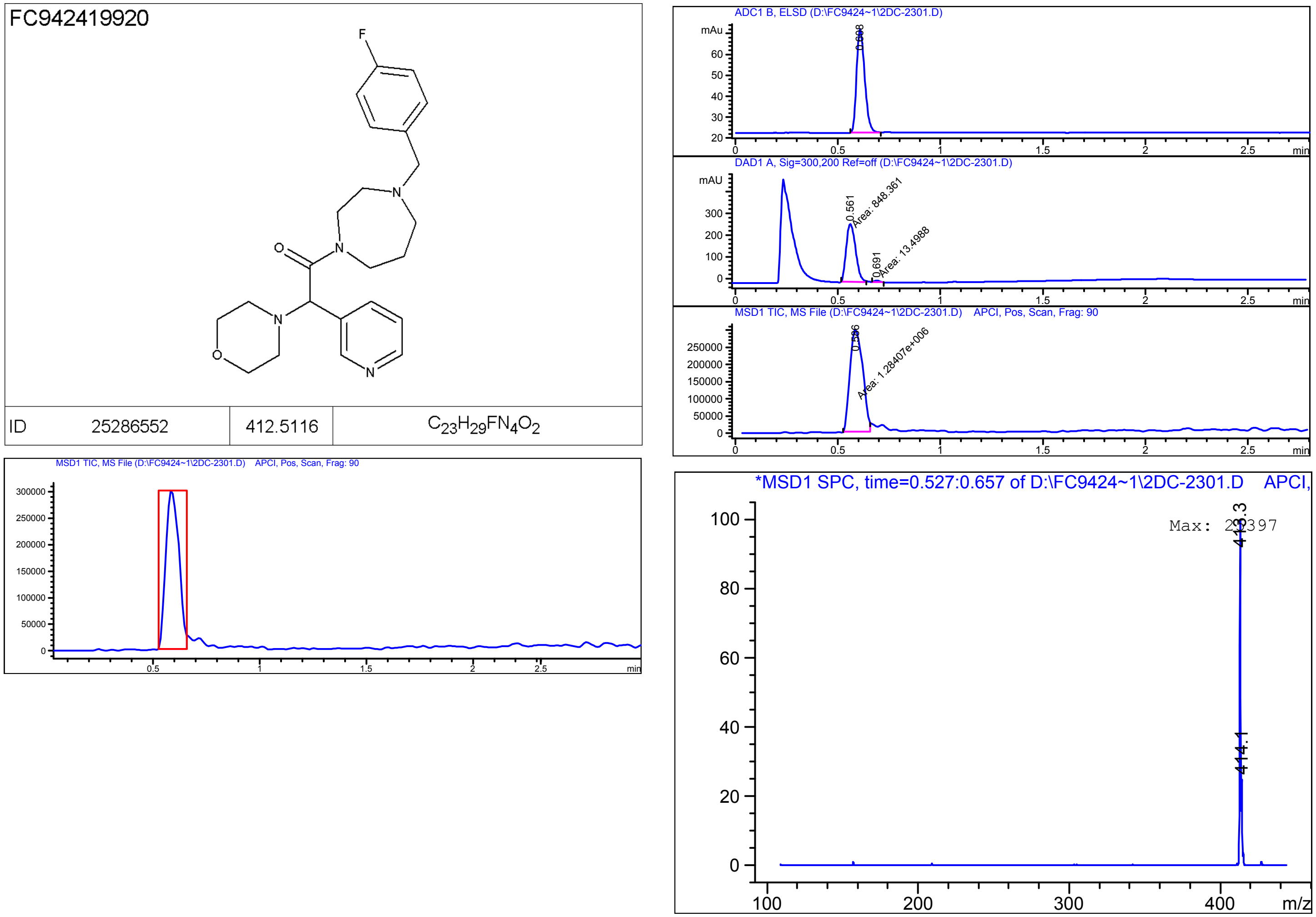

Figure S9. Chromatographic and mass spectra profiles obtained via LC-MS for 1-{4-[(4-fluorophenyl)methyl]-1,4-diazepan-1-yl}-2-(morpholin-4-yl)-2-(pyridin-3-yl)ethan-1-one (LC-65).

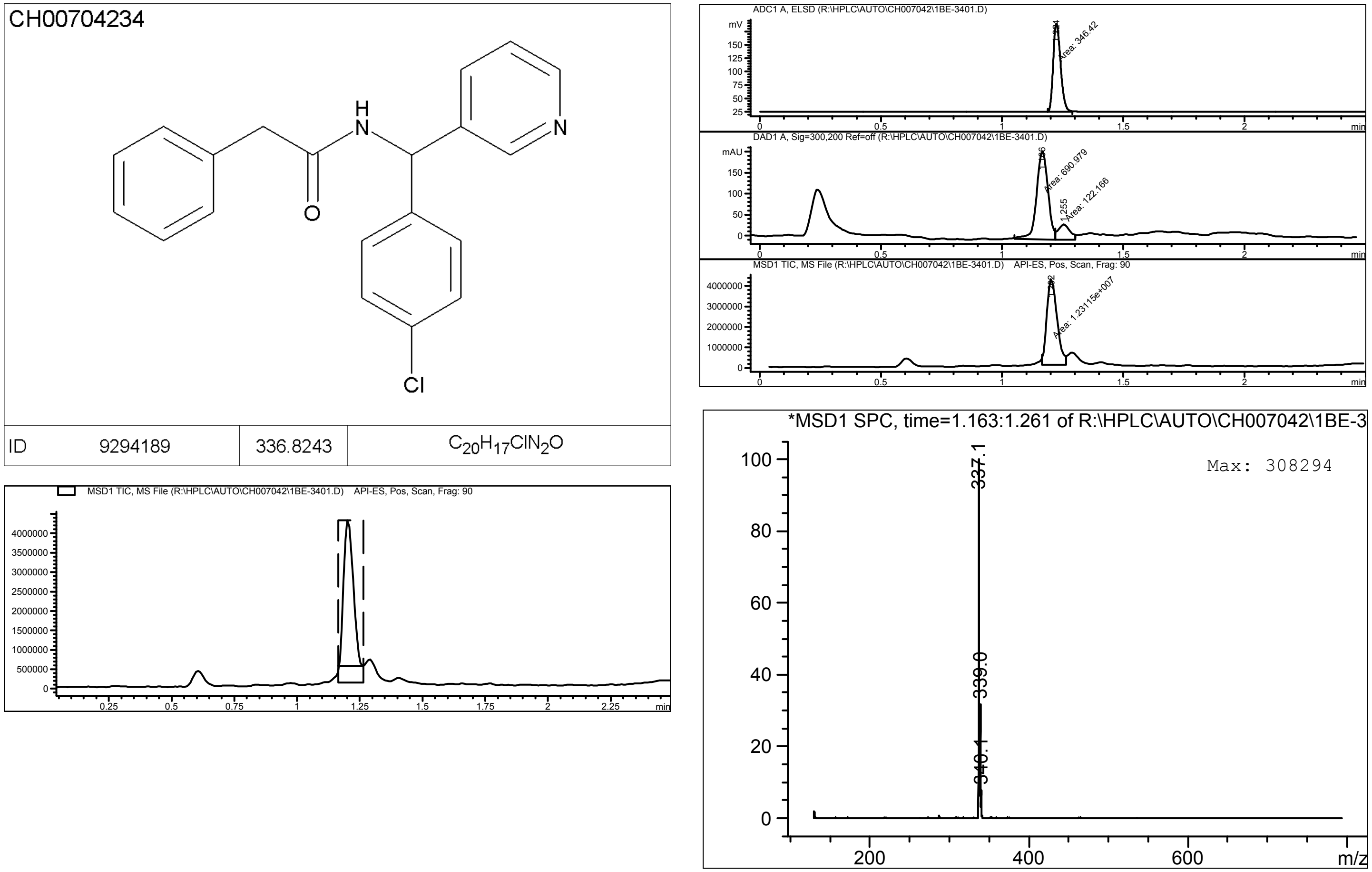

Figure S10. Chromatographic and mass spectra profiles obtained via LC-MS for N-[(4-chlorophenyl)(pyridin-3-yl)methyl]-2-phenylacetamide (LC-66).

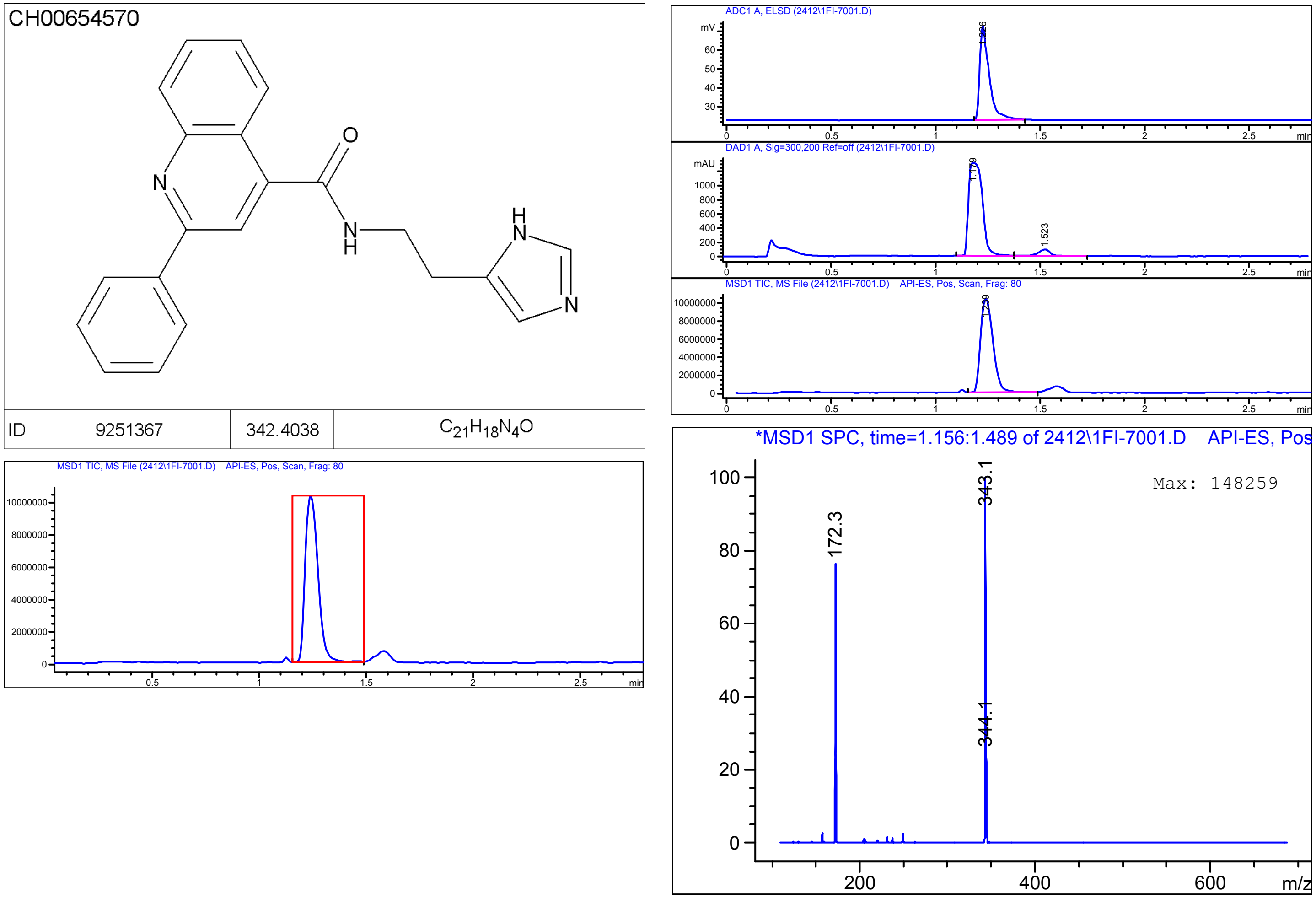

Figure S11. Chromatographic and mass spectra profiles obtained via LC-MS for N-[2-(1H-imidazol-5-yl)ethyl]-2-phenylquinoline-4-carboxamide (LC-67).

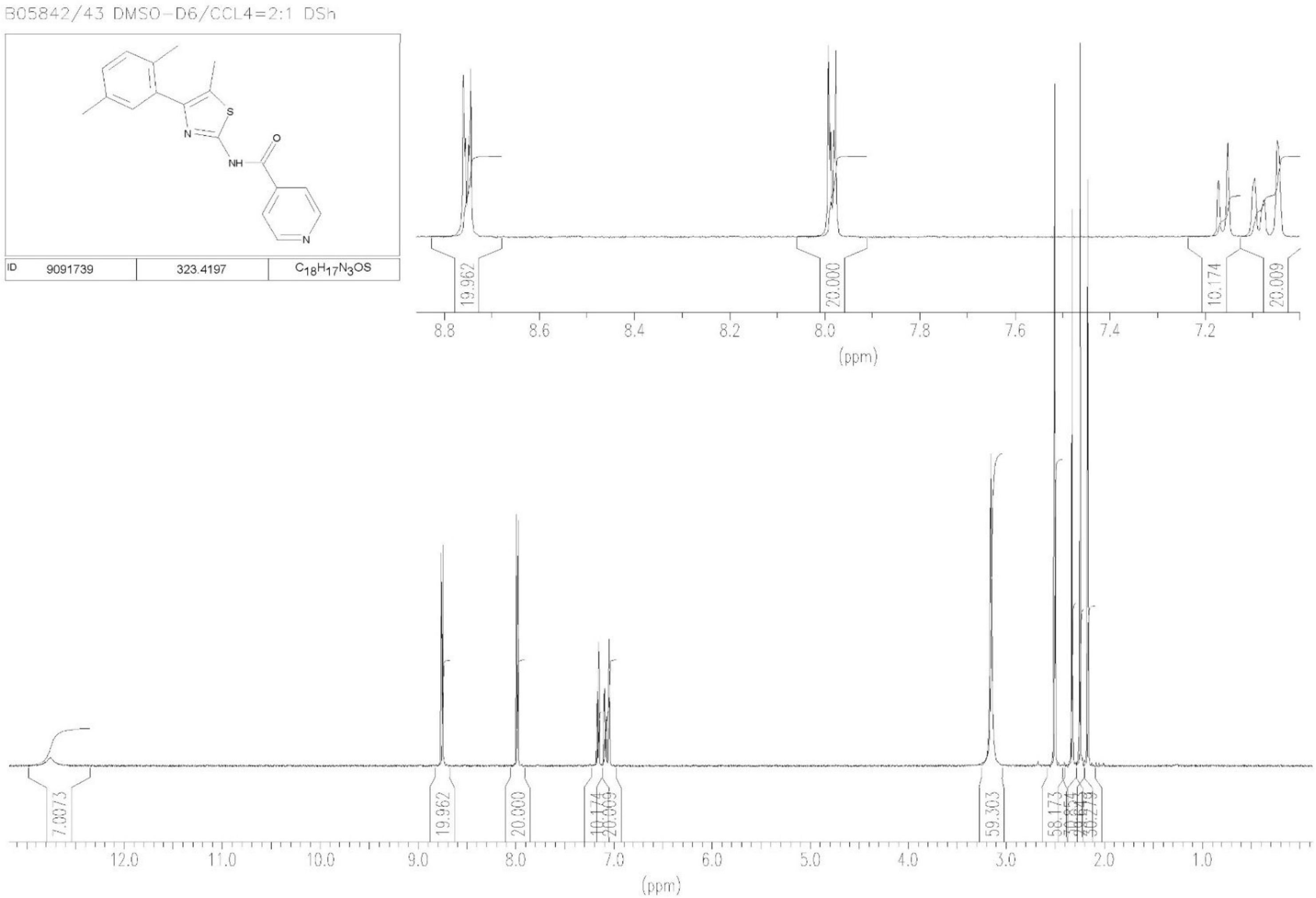

Figure S12. 1H NMR spectrum of compound N-[4-(2,5-dimethylphenyl)-5-methyl-1,3-thiazol-2-yl]pyridine-4-carboxamide (LC-68).

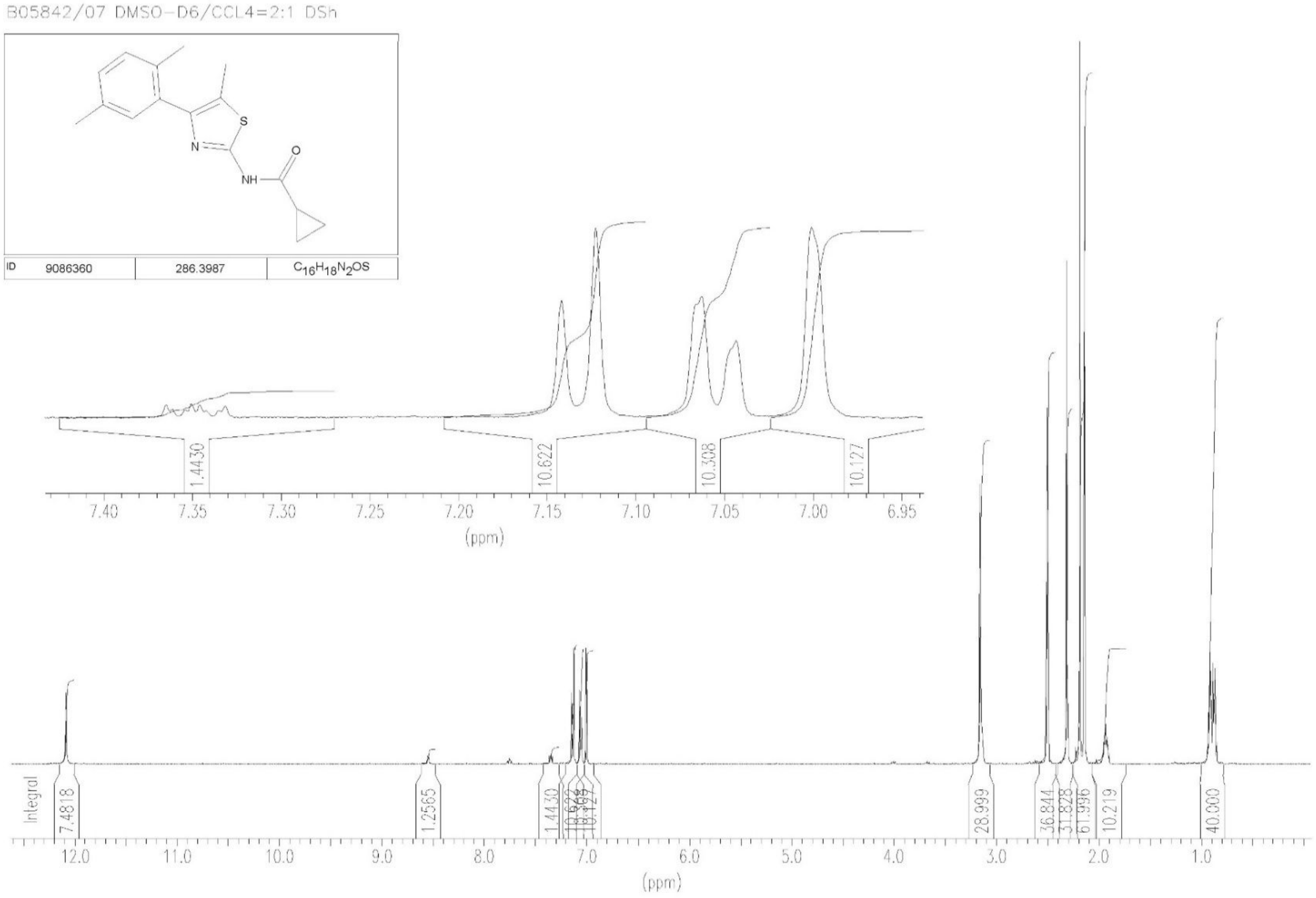

Figure S13. ^{1}H NMR spectrum of compound N-[4-(2,5-dimethylphenyl)-5-methyl-1,3-thiazol-2-yl]cyclopropanecarboxamide (LC-69).

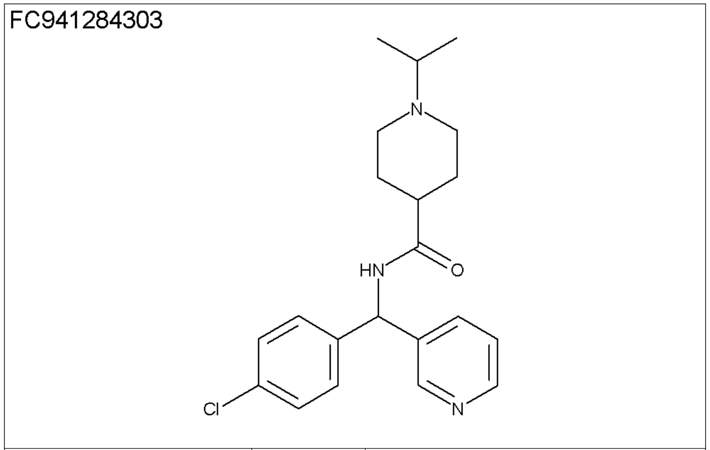

ID	39760868	371.9139	$C_{21}H_{26}ClN_3O$

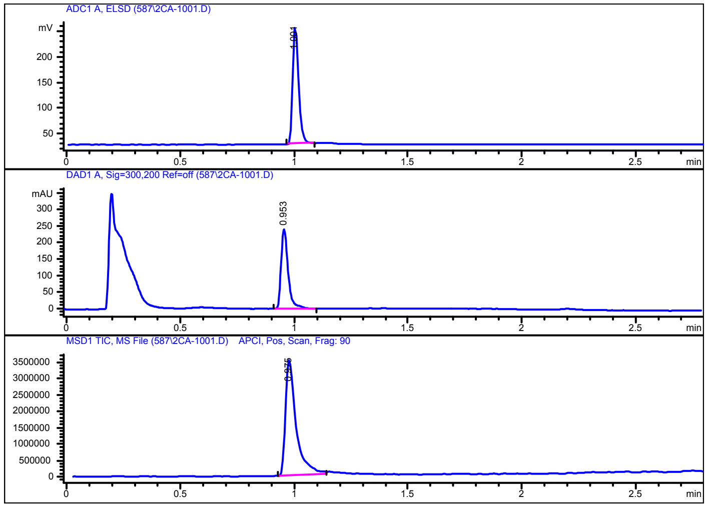

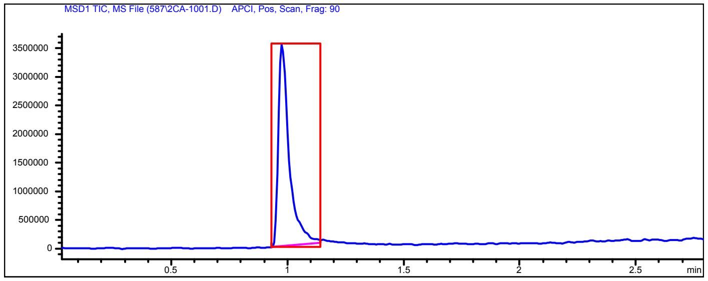

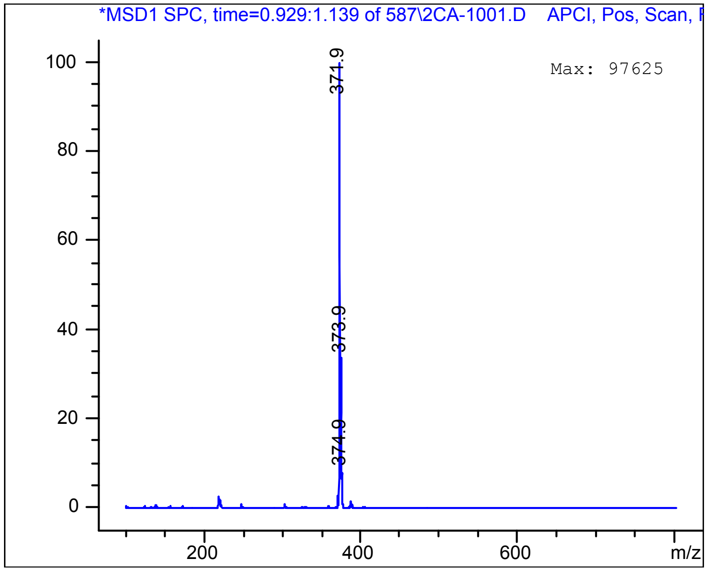

Figure S14. Chromatographic and mass spectra profiles obtained via LC-MS for N-[(4-chlorophenyl)(pyridin-3-yl)methyl]-1-(propan-2-yl)piperidine-4-carboxamide (LC-70).

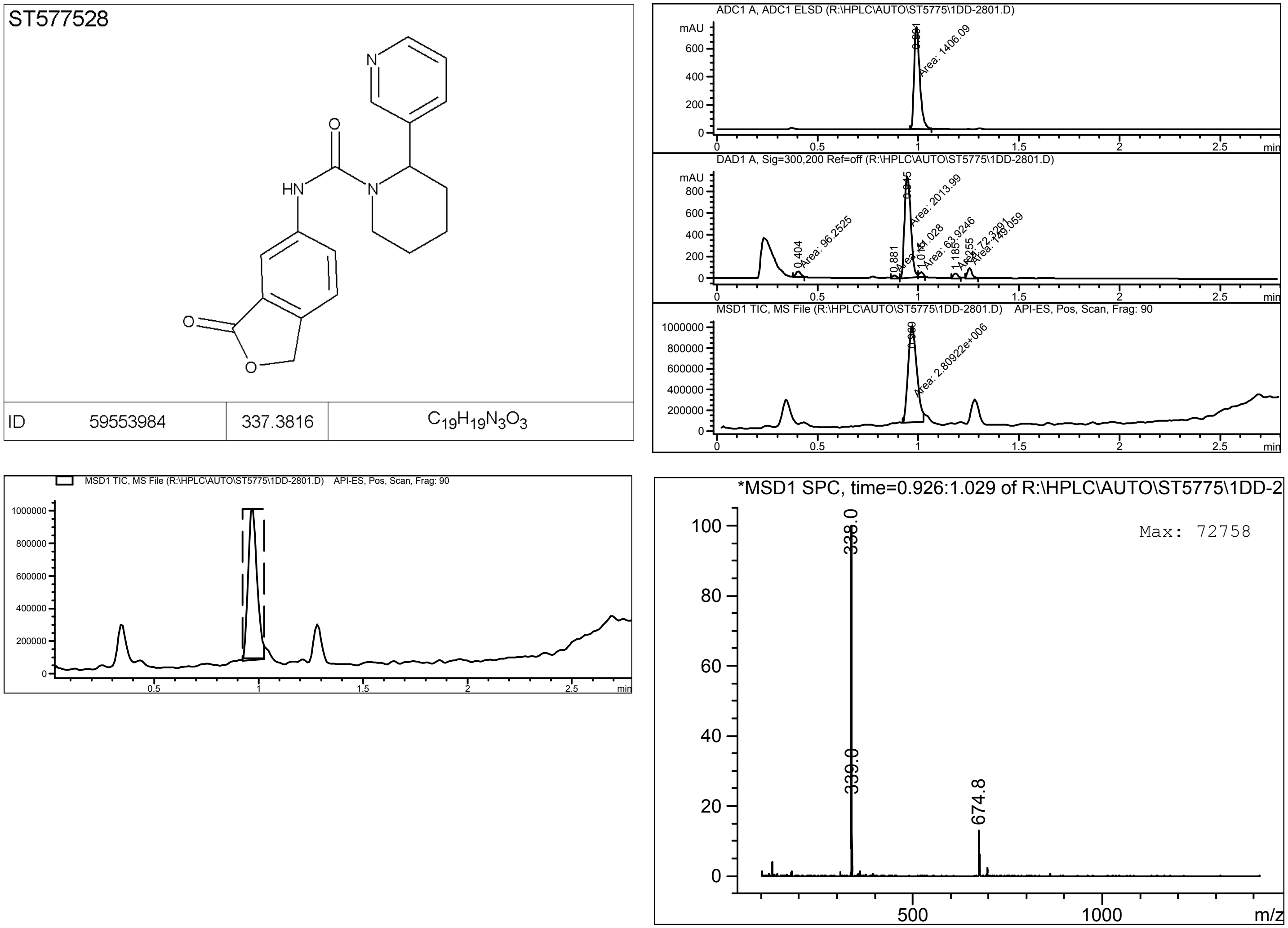

Figure S15. Chromatographic and mass spectra profiles obtained via LC-MS for N-(3-oxo-1,3-dihydro-2-benzofuran-5-yl)-2-(pyridin-3-yl)piperidine-1-carboxamide (LC-71).

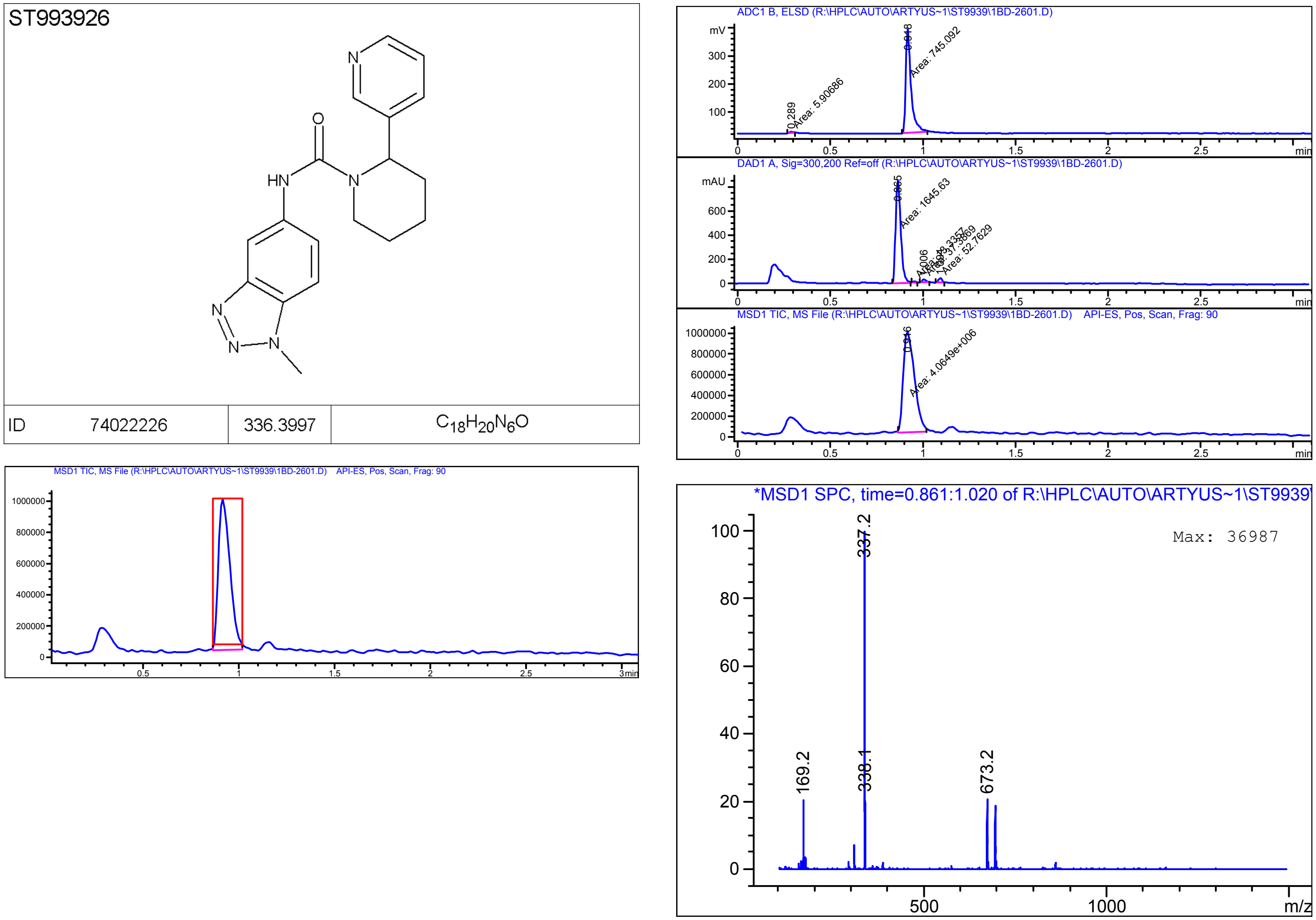

Figure S16. Chromatographic and mass spectra profiles obtained via LC-MS for N-(1-methyl-1H-1,2,3-benzotriazol-5-yl)-2-(pyridin-3-yl)piperidine-1-carboxamide (LC-72).

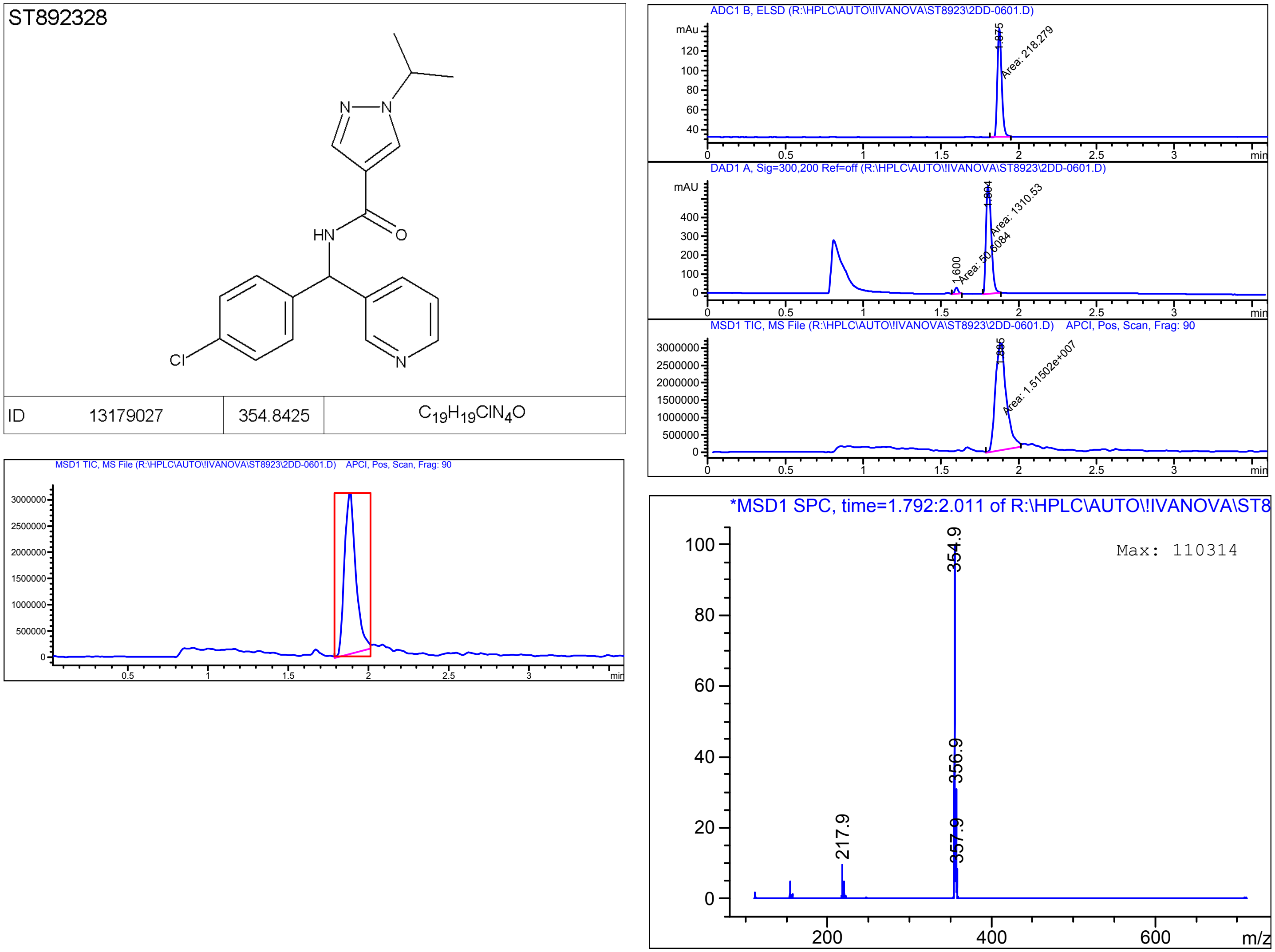

Figure S17. Chromatographic and mass spectra profiles obtained via LC-MS for N-[(4-chlorophenyl)(pyridin-3-yl)methyl]-1-(propan-2-yl)-1H-pyrazole-4-carboxamide (LC-73).

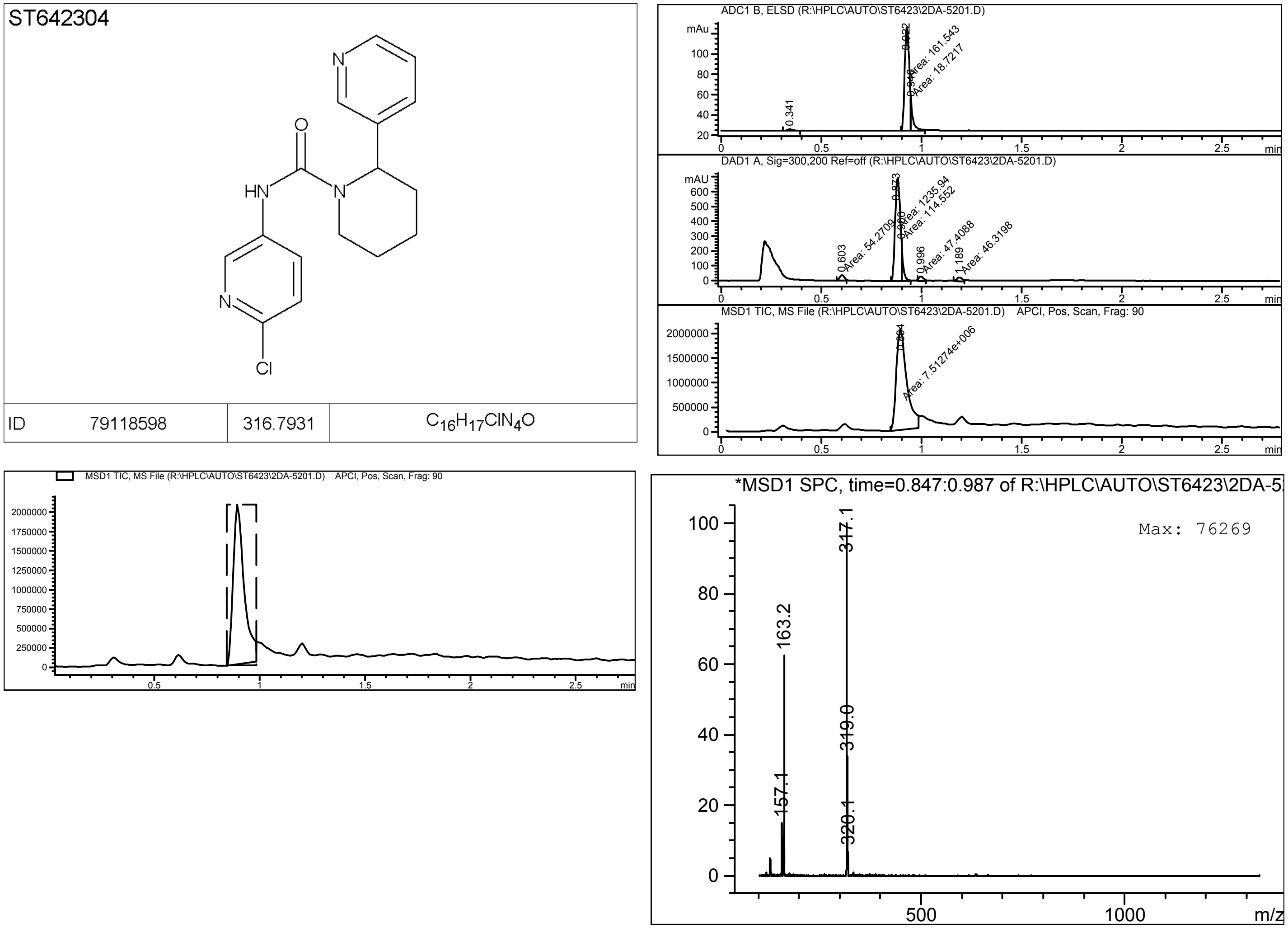

Figure S18. Chromatographic and mass spectra profiles obtained via LC-MS for N-(6-chloropyridin-3-yl)-2-(pyridin-3-yl)piperidine-1-carboxamide (LC-74).

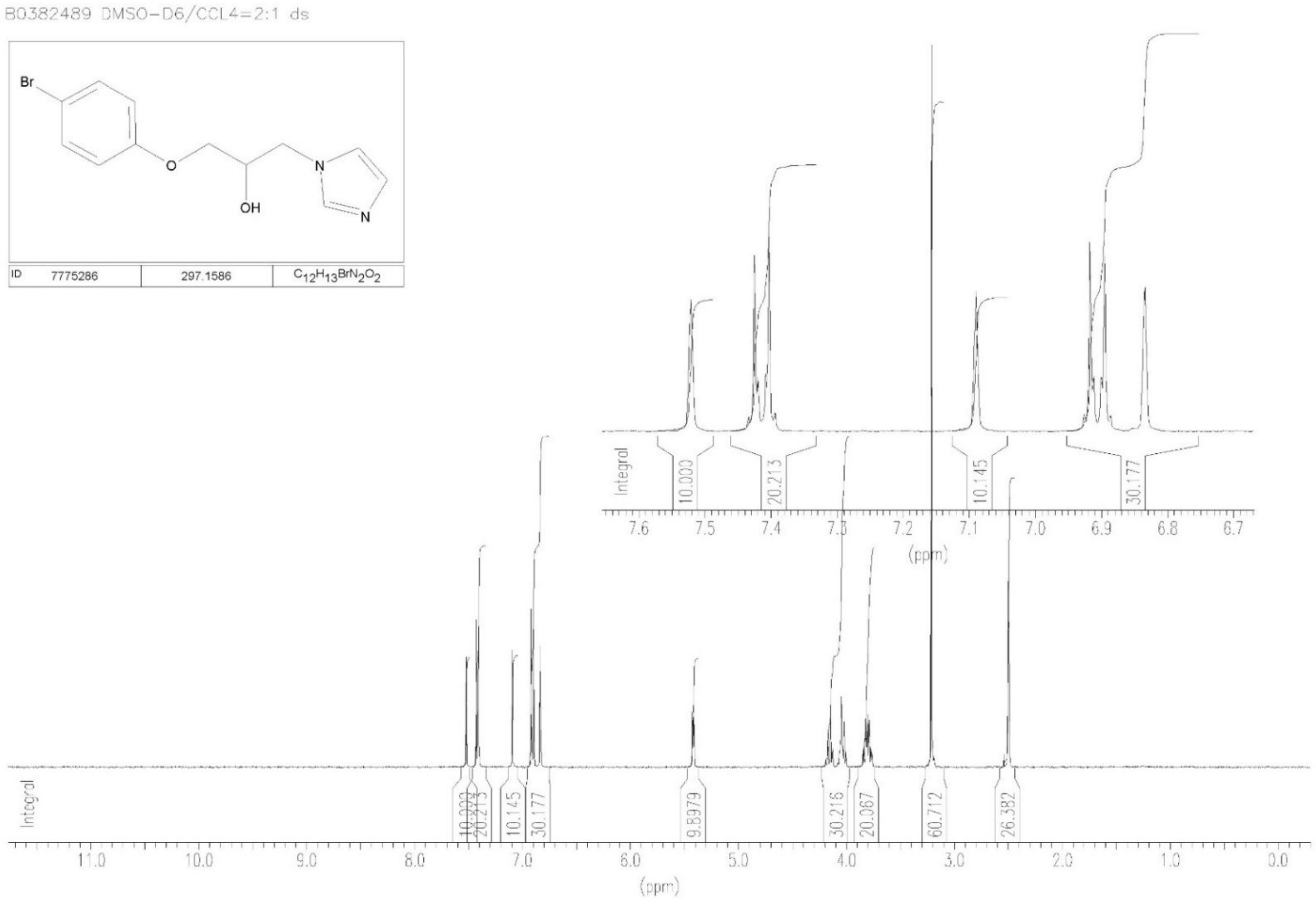

Figure S19. ^{1}H NMR spectrum of compound 1-(4-bromophenoxy)-3-(1H-imidazol-1-yl)propan-2-ol (LC-75).
